# Transgenic Tobacco Overexpressing *Brassica juncea* HMG-CoA Synthase 1 Shows Increased Plant Growth, Pod Size and Seed Yield

**DOI:** 10.1371/journal.pone.0098264

**Published:** 2014-05-21

**Authors:** Pan Liao, Hui Wang, Mingfu Wang, An-Shan Hsiao, Thomas J. Bach, Mee-Len Chye

**Affiliations:** 1 School of Biological Sciences, The University of Hong Kong, Hong Kong, China; 2 Centre National de la Recherche Scientifique, UPR 2357, Institut de Biologie Moléculaire des Plantes, Strasbourg, France; Lawrence Berkeley National Laboratory, United States of America

## Abstract

Seeds are very important not only in the life cycle of the plant but they represent food sources for man and animals. We report herein a mutant of 3-hydroxy-3-methylglutaryl-coenzyme A synthase (HMGS), the second enzyme in the mevalonate (MVA) pathway that can improve seed yield when overexpressed in a phylogenetically distant species. In *Brassica juncea,* the characterisation of four isogenes encoding HMGS has been previously reported. Enzyme kinetics on recombinant wild-type (wt) and mutant BjHMGS1 had revealed that S359A displayed a 10-fold higher enzyme activity. The overexpression of wt and mutant (S359A) BjHMGS1 in *Arabidopsis* had up-regulated several genes in sterol biosynthesis, increasing sterol content. To quickly assess the effects of BjHMGS1 overexpression in a phylogenetically more distant species beyond the Brassicaceae, wt and mutant (S359A) BjHMGS1 were expressed in tobacco (*Nicotiana tabacum* L. cv. Xanthi) of the family Solanaceae. New observations on tobacco OEs not previously reported for *Arabidopsis* OEs included: (i) phenotypic changes in enhanced plant growth, pod size and seed yield (more significant in OE-S359A than OE-wtBjHMGS1) in comparison to vector-transformed tobacco, (ii) higher *NtSQS* expression and sterol content in OE-S359A than OE-wtBjHMGS1 corresponding to greater increase in growth and seed yield, and (iii) induction of *NtIPPI2* and *NtGGPPS2* and downregulation of *NtIPPI1*, *NtGGPPS1*, *NtGGPPS3* and *NtGGPPS4*. Resembling *Arabidopsis* HMGS-OEs, tobacco HMGS-OEs displayed an enhanced expression of *NtHMGR1*, *NtSMT1-2*, *NtSMT2-1*, *NtSMT2-2* and *NtCYP85A1*. Overall, increased growth, pod size and seed yield in tobacco HMGS-OEs were attributed to the up-regulation of native *NtHMGR1*, *NtIPPI2*, *NtSQS*, *NtSMT1-2*, *NtSMT2-1*, *NtSMT2-2* and *NtCYP85A1*. Hence, S359A has potential in agriculture not only in improving phytosterol content but also seed yield, which may be desirable in food crops. This work further demonstrates HMGS function in plant reproduction that is reminiscent to reduced fertility of *hmgs* RNAi lines in *let-7* mutants of *Caenorhabditis elegans*.

## Introduction

Isoprenoids form a large and diverse group of natural products, which have promising pharmacological applications including anti-cancer, antibacterial and anti-malarial properties [1]–[4]. Some isoprenoids including gibberellic acids, abscisic acid, cytokinins, sterols and brassinosteroids (BRs) play significant roles in plant growth and development [4]–[6]. Furthermore, carotenoids and chlorophylls are involved in photosynthesis [7]. Phytosterols are important in regulating growth and mediating stress tolerance in plants [4], [8] and their nutritional value and health benefits in the human diet has been recognized [9]–[11].

**Figure 1:**
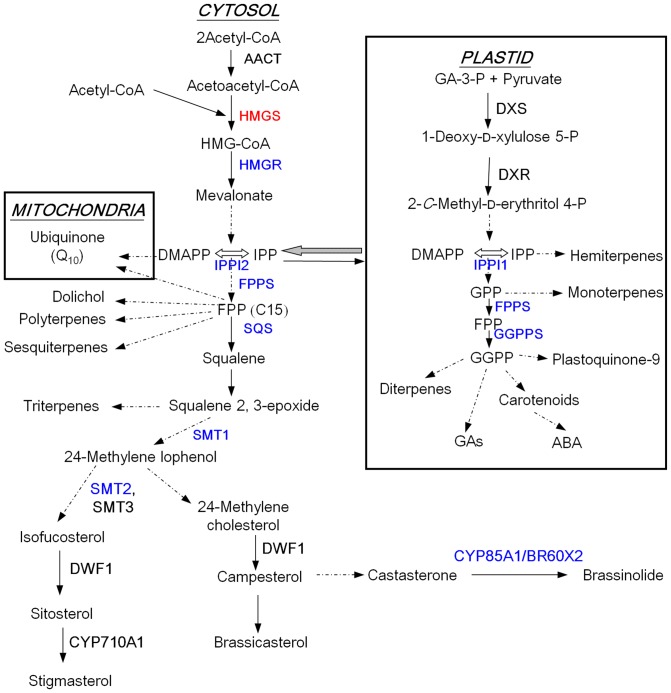
Outline of isoprenoid biosynthesis pathways in plants. Enzymes are shown in bold. Pathway inside the mitochondria and plastid are boxed. Arrows between cytosolic and plastid compartments represent metabolic flow between them (greater arrow for more flux). Abbreviations: ABA, abscisic acid; AACT, acetoacetyl-CoA thiolase; BR6OX2, brassinosteroid-6-oxidase 2; CYP710A1, sterol C-22 desaturase; CYP85A1, cytochrome P450 monooxygenase; DMAPP, dimethylallyl diphosphate; DWF1, delta-24 sterol reductase; DXR, 1-deoxy-D-xylulose 5-phosphate reductoisomerase; DXS, 1-deoxy-D-xylulose 5-phosphate synthase; FPP, farnesyl diphosphate; GA-3-P, glyceraldehyde-3-phosphate; FPPS, farnesyl diphosphate synthase; GAs, gibberellins; GGPP, geranylgeranyl diphosphate; GGPPS, geranylgeranyl diphosphate synthase; GPP, geranyl diphosphate; HMG-CoA, 3-hydroxy-3-methylglutaryl-CoA; HMGS, 3-hydroxy-3-methylglutaryl-CoA synthase; HMGR, 3-hydroxy-3-methylglutaryl-CoA reductase; IPP, isopentenyl diphosphate; IPPI, isopentenyl/dimethylallyl diphosphate isomerase; Q_10_, coenzyme Q_10_; SMT, sterol methyltransferase; SQS, squalene synthase. HMGS is marked in red colour. The expression levels of enzymes analysed in this work are marked in blue colour.

In higher plants, two pathways generate isopentenyl diphosphate (IPP), which constitutes the universal precursor of all isoprenoids: the mevalonate (MVA) pathway in the cytosol, and the non-MVA, methylerythritol phosphate (MEP) pathway in plastids [1], [3], [12](and references cited therein), with some crosstalk between them [13], [14] (Figure 1). Sterols and BRs are synthesized in the cytoplasm and thereby derive from MVA, while gibberellic acids and abscisic acid precursors, active cytokinins, carotenoids and chlorophylls are produced in plastids [1], [15]–[21] and thus depend on the MEP pathway (Figure 1).

In agriculture, it is desirable to increase seed yield because grains represent significant sources of food, and the relevant key genes must be identified. Plant isoprenoids including sterols and BRs are essential in plant growth and reproduction [6], [22]–[24] and genes from the BR-specific biosynthetic pathway, including *DWF4* and *DWF5*, affect seed production [22]–[24]. Transgenic *Arabidopsis* overexpressing *DWF4* showed better vegetative growth and seed yield [23], while the *Arabidopsis dwf5* mutant demonstrated a dwarf phenotype accompanied by abnormal seeds [22]. The genes in the first and third steps of the MVA pathway also affect plant growth and development. RNAi lines of *Arabidopsis* downregulated for cytoplasmic *ACETOACETYL-COA THIOLASE2* (*AACT2*) displayed reduction in apical dominance, seed yield and root length, accompanied by sterility and dwarfing [25]. Also, the *Arabidopsis hmgr1* mutant is dwarf-like and male sterile, and has a lower sterol content [26].

3-Hydroxy-3-methylglutaryl-coenzyme A synthase (HMGS) is the second enzyme in the MVA pathway [27]–[31]. Besides 3-hydroxy-3-methylglutaryl-coenzyme A reductase (HMGR), HMGS is a key enzyme in cholesterol biosynthesis in mammals and cytoplasmic isoprenoid biosynthesis in plants [3], [4], [32]–[36]. Four genes designated *BjHMGS1 to BjHMGS4* encode HMGS in *Brassica juncea* [34] and investigations revealed that BjHMGS1 is cytosolic. The expression of recombinant BjHMGS1 led to the elucidation of its kinetic and physiological properties [37], [38] and of its crystal structure [39]. Enzyme kinetics of recombinant wild-type (wt) and mutant BjHMGS1 had revealed that H188N showed 8-fold lower enzyme activity and loss of acetoacetyl-CoA inhibition, while S359A displayed a 10-fold higher enzyme activity [37]. Given these interesting results, mutant (H188N, S359A and H188N/S359A) and wt BjHMGS1 were overexpressed in *Arabidopsis*, which like *Brassica*, belongs to the family Brassicaceae [4]. *BjHMGS1* overexpression in transgenic *Arabidopsis* up-regulated several genes in sterol biosynthesis (cf. Figure 1), for instance those encoding HMGR, SMT2 (sterol methyltransferase 2), DWF1 (sterol C-24-reductase), CYP710A1 (sterol C-22 desaturase) and BR6OX2 (brassinosteroid-6-oxidase 2), increasing sterol content and thereby enhancing stress tolerance [4]. Analysis of the *Arabidopsis hmgs* mutant demonstrated the role of HMGS in tapetal development and pollen fertility [35].

To quickly assess the effects of BjHMGS1 overexpression in a more distant species, the overexpression of BjHMGS1 was carried out on a plant outside the Brassicaceae family. Hence, tobacco (*Nicotiana tabacum* L. cv. Xanthi), another model plant from the family of Solanaceae was selected, also because of the easiness of its genetic transformation. Subsequently, the genes downstream of *HMGS* that were tested encode enzymes that produce intermediates in phytosterol and BR biosynthesis, for instance *N. tabacum* 3-hydroxy-3-methylglutaryl-CoA reductase (NtHMGR1 and NtHMGR2), isopentenyl diphosphate isomerase (NtIPPI1 and NtIPPI2), farnesyl diphosphate synthase (NtFPPS), squalene synthase (NtSQS), sterol methyltransferases (NtSMT1-2, NtSMT2-1 and NtSMT2-2) and cytochrome P450 monooxygenase (NtCYP85A1). In addition, we examined the expression of genes encoding geranylgeranyl diphosphate synthases (NtGGPPS1, NtGGPPS2, NtGGPPS3 and NtGGPPS4), enzymes that are not implied in the formation of an intermediate in the sterol pathway. Resultant transgenic tobacco (OE-wtBjHMGS1 and OE-S359A) not only showed an increased sterol content but also displayed enhanced plant growth, pod size and seed yield that were not previously observed in transgenic *Arabidopsis* HMGS-OEs. Furthermore, OE-S359A conferred better plant growth and seed production than OE-wtBjHMGS1, and this was attributed to higher *NtSQS* expression and total sterol content, realizing the potential application of *BjHMGS1* in being quite active in phylogenetically distant species.

## Materials and Methods

### Plant materials and growth conditions

Wt tobacco (*N. tabacum* L. cv. Xanthi) obtained from the Institute of Molecular and Cell Biology (Singapore) was used in this study. Tobacco plants were grown at 25°C (16 h light)/22°C (8 h dark). Tobacco seedlings were cultured in Murashige and Skoog (MS) medium [40].

### Generation of transgenic plants overexpressing HMGS

Plasmids pBj134 (wtBjHMGS1) and pBj136 (S359A) were used in *Agrobacterium*-mediated leaf disc transformation of *N. tabacum* [4], [41]. The binary vector pSa13 [42] was used as vector control in transformation. T_1_ transgenic tobacco seeds were selected on MS containing kanamycin (50 µg ml^−1^) and verified using PCR and DNA sequence [4]. T_2_ homozygous plants with a single-copy transgene were compared in mRNA expression, metabolite composition, plant growth and seed yield.

### Western blot analysis

Total protein was extracted [43] from 21-d-old tobacco leaves. Protein concentration was determined using the Bio-Rad Protein Assay Kit I (Bio-Rad). Protein (20 µg per well) separated on 12% SDS-PAGE was transferred onto Hybond-ECL membrane (Amersham) using a Trans-Blot^®^ cell (Bio-Rad). Antibodies raised against the synthetic peptide (DESYQSRDLEKVSQQ) corresponding to BjHMGS1 amino acids 290 to 304 were used in western blot analyses [4], [44]. Cross-reacting bands were detected using the ECL™ Western Blotting Detection Kit (Amersham).

### Northern blot analysis

Tobacco total RNA was extracted from 21-d-old tobacco leaves using TRIzol reagent (Invitrogen). RNA (20 µg per well), separated on 1.3% agarose gels containing 6% formaldehyde, was transferred to Hybond-N membrane (Amersham) for northern blot analysis [45]. Digoxigenin-labelled probes were synthesized using the PCR Digoxigenin Probe Synthesis (Roche) with primer pairs ML276 and ML860 for *BjHMGS1*. Primers are listed in Table S1.

### Southern blot analysis

Genomic DNA (40 µg) from 4-week-old tobacco leaves prepared by the CTAB method [46] was digested by *Eco*RI and separated on 0.7% agarose gel by electrophoresis, together with a 1-kb plus DNA standard ladder (Invitrogen). DNA was transferred from the agarose gel onto Hybond-N membrane (Amersham) by capillary transfer [47]. Southern blot analysis of tobacco using a ^32^P-labelled full-length of *BjHMGS1* cDNA probe with primer pair ML264 and ML860 was performed [4]. Primers are listed in Table S1.

### Extraction and quantitative analysis of sterols

For sterol profiling, freeze-dried materials from 20 mg of 60-d-old soil-grown tobacco leaves and 10 mg of 20-d-old MS plate-cultured tobacco seedlings were used. Extraction and quantitative analysis of sterols were carried out as described [4], [48]. GC-MS analysis (GC: Hewlett Packard 6890 with an HP-5MS capillary column: 30 m long, 0.25 mm i.d., film thickness 0.25 µm; MS: Hewlett Packard 5973 mass selective detector, 70 eV) was used to determine sterol content, with He as the carrier gas (1 ml/min). The column temperature program used included a fast rise from 60°C to 220°C (30°C/min) and a slow rise from 220°C to 300°C (5°C/min), then kept at 300°C for 10 min. The inlet temperature was 280°C. Compounds were identified using the National Institute of Standards and Technology (NIST) libraries of peptide tandem mass spectra (Agilent, USA). The sterol masses were determined by comparison of the peak area of each compound with that of the internal standard (lupenyl-3,28-diacetate). Two independent lines for each OE genotype were analysed. Five independent repeats (samples) for each independent line were used for sterol extraction. Each sample was injected twice in GC-MS analyses and an average of the sterol mass was taken. Sitosterol, campesterol and stigmasterol contents in transgenic tobacco HMGS-OEs were compared to those in vector (pSa13)-transformed plants following previous reports [4], [17].

### Seed germination assay

Tobacco seeds collected simultaneously from vector (pSa13)-transformed control and HMGS-OE lines were sterilized in 20% bleach, 70% ethanol and then spread on MS medium agar plates supplemented with kanamycin (50 mg/l). About 30 tobacco seeds were sown on one plate. Five duplicate plates were used for each independent line [4]. All the plates were incubated at 4°C for 4 days and transferred to a culture room for 2 days under a photoperiod of 22°C 8-h dark and 23°C 16-h light. Subsequently, the number of germinated seeds was counted every 12 h for 60 h using a dissecting microscope. The emergence of the radicle was defined as germination [4]. The germination rates were calculated and compared using the Student's *t*-test. Two independent lines of OE-wtBjHMGS1 (“401” and “402”) and two independent lines of OE-S359A (“603” and “606”) were tested in seed germination assays. The experiment to measure seed germination was repeated twice.

### Growth rate measurements

Growth rate was measured according to previous reports [49]–[53]. Four-d-old seedlings were transferred onto fresh MS plates placed vertically for a further 10-d growth. The dry weight of 14-d-old seedlings was then measured. Five seedlings were grouped for weight measurements and a total of 30 groups were analysed per individual line.

For greenhouse plants, 7-d-old tobacco seedlings of similar size were transferred from MS medium to soil for further growth rate measurements. The height of 80-, 98- and 210-d-old tobacco were measured. As 80-d-old plants did not have flowers, the height measurement did not include the inflorescence. However, 98- and 210-d-old plants were flowering and the height measurement included the inflorescence. For 98-d-old tobacco, measurements of leaf fresh weight, length and width of the four bottom-most leaves were also analysed for the vector-transformed control, OE-wtBjHMGS1 and OE-S359A. Two independent lines from each OE construct were analysed for 80-d-old tobacco plants and three independent lines from each OE construct were analysed for 98- and 210-d-old tobacco plants. For each line, six plants were used.

### Comparison in tobacco seed yield

Seed yield was measured [49], [51], [52], [54] to test the differences between HMGS-OEs (OE-wtBjHMGS1 and OE-S359A) and the vector-transformed control. Ten plants each from two independent lines from each OE construct were examined and T_2_ homozygous seeds of each line were germinated on MS. Fourteen-d-old seedlings were transferred to soil in a greenhouse. Pods (30 per group) were harvested at maturity from each of 10 plants per line to determine total dry pod weight, average dry pod weight, total dry seed weight and total seed number. The experiment to measure seed yield was repeated twice (2–3 groups were analysed for each repeat).

To further determine if increase in seed size occurred, the dry weight of 100 seeds from each line was measured and 29 repeats were carried out per line. The average dry weight was calculated from 30 measurements of 100 seeds per line.

### RNA analysis

Total RNA (5 µg) of 20-d-old tobacco seedlings and 14-d-old Arabidopsis were extracted using RNeasy Plant Mini Kit (Qiagen) and were reverse-transcribed into first-strand cDNA using the SuperScript First-Strand Synthesis System (Invitrogen). Quantitative Reverse Transcription-PCR (qRT-PCR) was carried out with a StepOne Plus Real-time PCR System (Applied Biosystems) and FastStart Universal SYBR Green Mater (Roche). The conditions for qRT-PCR were as follows: denaturation at 95°C for 10 min, followed by 40 cycles of 95°C for 15 s and 60°C for 1 min. Three experimental replicates for each reaction were carried out using gene-specific primers and tobacco *ACTIN* and Arabidopsis *ACTIN2* were used as internal controls. The relative changes in expression from three independent experiments were analysed [55]. Primers for qRT-PCR are listed in Table S1.

### Accession numbers

Sequence data included herein can be found in the GenBank/EMBL data libraries under accession numbers AF148847 (*BjHMGS1*), AY140008 (*AtHMGS*), U60452 (*NtHMGR1*), AF004232 (*NtHMGR2*), AB049815 (*NtIPI1*), AB049816 (*NtIPI2*), GQ410573 (*NtFPPS*), U60057 (*NtSQS*), GQ911583 (*NtGGPPS1*), GQ911584 (*NtGGPPS2*), AF053766 (*NtSMT1-2*), U71108 (*NtSMT2-1*), U71107.1 (*NtSMT2-2*), DQ649022 (*NtCYP85A1*), U60489 (*NtACTIN*), BT003419 (*AtSQS*) and AY096381 (*AtACTIN2*).

### Statistical analysis

Analyses of data in this work was carried out using the Student's *t*-test to determine any significant differences between means.

## Results

### Molecular analyses of transgenic tobacco HMGS-OEs

**Figure 2:**
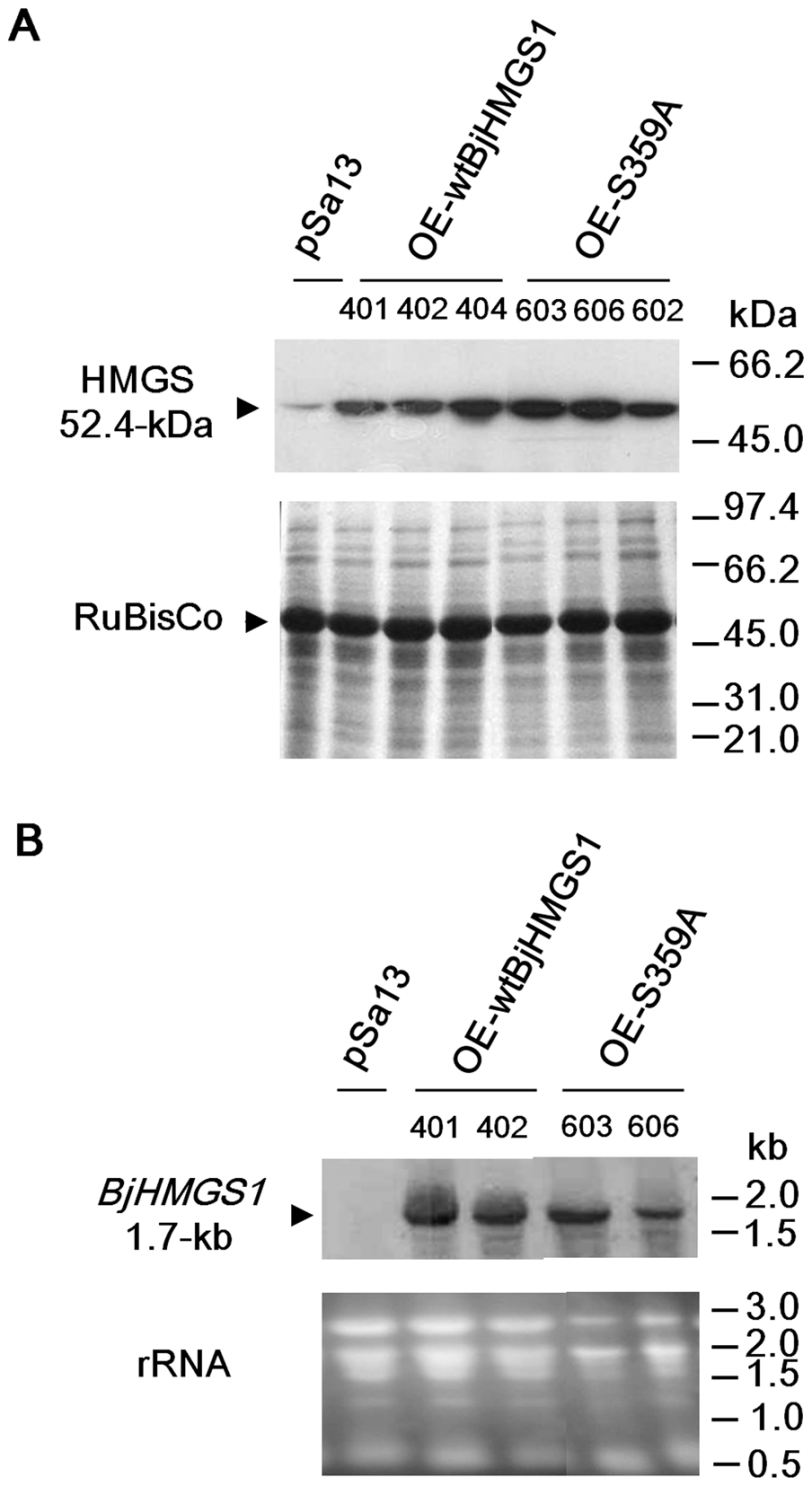
Molecular analysis of representative transgenic tobacco HMGS-OEs. (A) Western blot analysis using antibodies against BjHMGS1 to verify the expression of BjHMGS1 (52.4-kDa) in representative vector (pSa13)-transformed control and HMGS-OEs (OE-wtBjHMGS1 and OE-S359A). Putative tobacco HMGS-OEs were designated as OE-wtBjHMGS1 (lines “401”, “402” and “404”) and OE-S359A (lines “602”, “603” and “606”). Bottom, Coomassie Blue-stained gel of total protein loaded (20 µg per well). Three independent lines per construct were analysed. (B) Northern blot analysis of *BjHMGS1* in representative vector (pSa13)-transformed control and HMGS-OEs. The expected 1.7-kb *BjHMGS1* band is marked with an arrowhead. Bottom gels show rRNA (20 µg per lane). Two independent lines per construct are shown. The two independent lines of OE-wtBjHMGS1 plants labelled “401” and “402”, and two independent lines of OE-S359A plants labelled “603” and “606” used in further tests are underlined.

The presence of wt and mutant *BjHMGS1* in transgenic tobacco was verified by PCR (Figure S1A-B) followed by DNA sequence analysis of the PCR product. Putative tobacco HMGS-OEs were designated as OE-wtBjHMGS1 (lines “401”, “402” and “404”) and OE-S359A (lines “602”, “603” and “606”). PCR-positive HMGS-OE lines were confirmed by western blot analysis (Figure 2A). As the peptide used to generate anti-BjHMGS1 antibodies shows 100% homology to tobacco HMGS (GenBank accession number EF636813), a faint band was detected in the vector (pSa13)-transformed control (Figure 2A). Northern blot analyses revealed that transgenic lines verified by western blot analysis expressed *BjHMGS1* mRNA (Figure 2B). Single-insertional lines identified by Southern blot analyses (Figure S2) were selected for further experiments.

### Tobacco HMGS-OEs accumulate sterols in both seedlings and leaves

The contents of the three major sterols (campesterol, stigmasterol and sitosterol) in 20-d-old tobacco HMGS-OE seedlings and 60-d-old leaves were analysed. GC-MS results of changes represented in µg per mg dry weight showed that the average campesterol, stigmasterol, sitosterol and total sterol contents of the OE-S359A seedlings were significantly higher than the vector (pSa13)-transformed control and OE-wtBjHMGS1 (Table 1). In particular, the average elevations over the vector (pSa13)-transformed control in OE-S359A seedlings were noted for campesterol (31.7%), stigmasterol (24.0%), sitosterol (25%) and total sterol (25.7%) (Table 2) and average elevations over OE-wtBjHMGS1 for campesterol (25.4%), stigmasterol (19.0%), sitosterol (20%) and total sterol (20.4%) (Table 2). However, OE-wtBjHMGS1 seedlings did not show significant changes from the vector-transformed control and increases were merely ∼4–5% for each sterol (Table 2).

**Table 1:** Sterol profiles of tobacco HMGS-OE seedlings and leaves ( µg/mg dry weight).

Construct	Sterol content of 20-d-old seedlings				Sterol content of 60-d-old leaves			
	Campesterol	Stigmasterol	Sitosterol	Total sterol	Campesterol	Stigmasterol	Sitosterol	Total sterol
**pSa13**	0.60±0.08	1.21±0.15	0.48±0.08	2.49±0.29	0.85±0.06	0.66±0.05	0.14±0.02	1.74±0.10
**401**	0.64±0.05	1.25±0.04	0.51±0.02	2.59±0.07	**0.98±0.05^a^**	0.68±0.04	**0.21±0.01^a^**	**1.99±0.08^a^**
**402**	0.63±0.03	1.26±0.06	0.49±0.02	2.61±0.11	**0.93±0.04^a^**	0.73±0.02	**0.18±0.01^a^**	**1.91±0.05^a^**
**603**	**0.79±0.04^a,b^**	**1.50±0.04^a,b^**	**0.59±0.02^a,b^**	**3.14±0.10^a,b^**	0.87±0.03	**0.91±0.05^a^**	0.16±0.02	**2.12±0.06^a^**
**606**	**0.79±0.03^a,b^**	**1.49±0.05^a,b^**	**0.61±0.02^a,b^**	**3.12±0.09^a,b^**	0.89±0.05	**0.82±0.04^a^**	0.16±0.01	**2.01±0.04^a^**

Two independent lines for each OE genotype were analysed. For OE-wtBjHMGS1, transformants “401” and “402” were tested. For OE-S359A, transformants “603” and “606” were tested. a indicates significant difference between HMGS-OE and the vector (pSa13)-transformed control; b indicates significant difference between OE-wtBjHMGS1 and OE-S359A. Bold font indicates significant higher sterol content than vector (pSa13)-transformed control and/or the OE-wtBjHMGS1 (*P*<0.01 by the Student's *t*-test). Values are mean ±SD, n = 5.

**Table 2:** Increase (%) of sterol composition in tobacco HMGS-OE seedlings and leaves in comparison to vector (pSa13)-transformed control and elevation of OE-S359A over OE-wtBjHMGS1.

Construct	Compared to	Elevation (%) in 20-d-old seedlings				Elevation (%) in 60-d-old leaves			
		Campesterol	Stigmasterol	Sitosterol	Total sterol	Campesterol	Stigmasterol	Sitosterol	Total sterol
**401**	pSa13	6.7	3.3	6.3	4.0	15.3	3.0	50.0	14.4
**402**	pSa13	5.0	4.1	2.1	4.8	9.4	10.6	28.6	9.8
**603**	pSa13	**31.7**	**24.0**	**22.9**	**26.1**	2.4	**37.9**	14.3	**21.8**
**606**	pSa13	**31.7**	**23.1**	**27.1**	**25.3**	4.7	**24.2**	14.3	**15.5**
OE-S359A	OE-wtBjHMGS1	**25.4**	**19.0**	**20.0**	**20.4**	−8.3	**24.3**	−20.0	6.2

Two independent lines for each OE genotype were analysed. For tobacco OE-wtBjHMGS1, transformants “401” and “402” were tested. For tobacco OE-S359A, transformants “603” and “606” were tested. Values  =  [(mean_OEs_ - mean_pSa13_)/mean_pSa13_]*100. The data presented for OE-S359A in comparison to OE-wtBjHMGS1 was calculated from an average of two transformants (average of “603” and “606” for OE-S359A in comparison to average of “401” and “402” for OE-wtBjHMGS1). Bold font indicates % increase value in OE-S359A which was higher than the corresponding OE-wtBjHMGS1.

In leaves, except for stigmasterol, the average amounts of campesterol, sitosterol and total sterol were significantly higher in OE-wtBjHMGS1 than the vector (pSa13)-transformed control (Table 1): campesterol (12.9%), sitosterol (42.9%) and total sterol (12.1%) (Table 2). Furthermore, the average amounts of stigmasterol and total sterol in OE-S359A leaves were significantly higher (31.8% and 19.0%, respectively) over the vector (pSa13)-transformed control (Table 2). The differences between OE-wtBjHMGS1 and OE-S359A leaves were not significant and OE-S359A average stigmasterol and total sterol contents were only slightly higher than OE-wtBjHMGS1 (Table 1).

The % increase of sterols between transgenic tobacco (observed herein) and transgenic Arabidopsis (OE-wtBjHMGS1 and OE-S359A) [4] were also compared (Table 2 and S2). A similar trend was observed in transgenic Arabidopsis and tobacco seedlings; OE-S359A transformants displayed higher increase than the OE-wtBjHMGS1 not only in each sterol (campesterol, stigmasterol and sitosterol) but also in total sterol (Table S2). OE-S359A transformants also showed similar increase over the OE-wtBjHMGS1 in both Arabidopsis and tobacco leaves for stigmasterol and total sterol (Table 2 and S2).

### Tobacco HMGS-OE seeds germinated earlier

As seeds from Arabidopsis HMGS-OEs were observed to germinate earlier than the vector (pSa13)-transformed control [4], the germination of tobacco HMGS-OE seeds was investigated. Tobacco seeds of OE-wtBjHMGS1 and OE-S359A not only germinated earlier but also displayed significantly higher germination rates than the control at 60 to 120 h post-germination (Figure S3). Also, OE-S359A germinated faster than OE-wtBjHMGS1 (Figure S3).

### Tobacco HMGS-OE plants show increased growth

**Figure 3:**
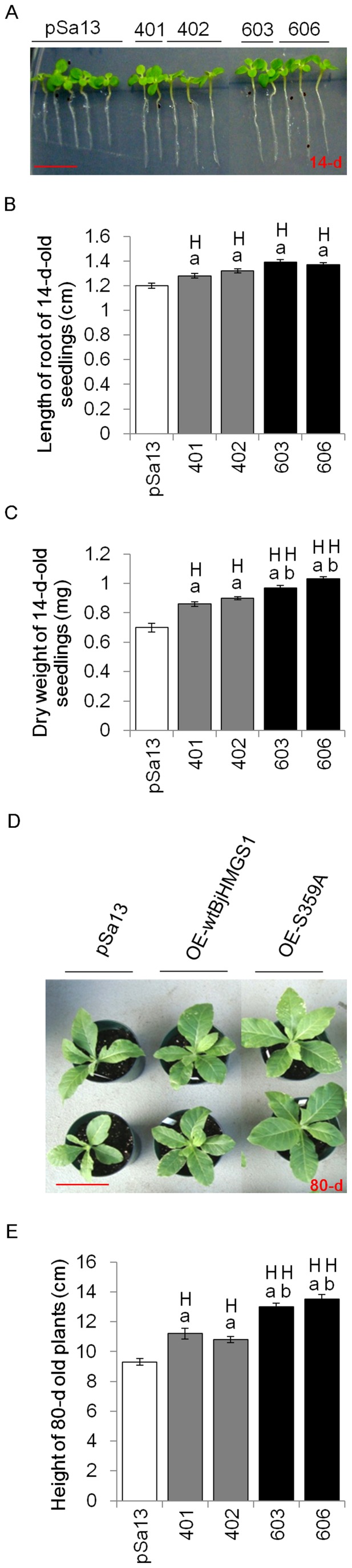
Comparison in growth between tobacco HMGS-OE seedlings/plants and vector-transformed control. (A) Seedlings 14-d post-germination. The vector-transformed control is labelled “pSa13”, two independent lines of OE-wtBjHMGS1 plants are labelled “401” (two representative seedlings of this OE construct were shown) and “402” (three representative seedlings of this OE construct were shown) and two independent lines of OE-S359A plants are labelled “603” (two representative seedlings of this OE construct were shown) and “606” (three representative seedlings of this OE construct were shown). Bar  = 1 cm. (B) Root length measurements of 14-d-old seedlings showed that tobacco HMGS-OE roots grow faster than the vector (pSa13)-transformed control. Values are mean ±SD (n = 30); Bars are SD. (C) Dry weight determination of 14-d-old seedlings shows that tobacco HMGS-OEs possess a higher mass than the vector-transformed control. Values are mean ± SD (n = 30); Bars are SD. (D) Representative greenhouse-grown plants photographed 80-d after germination. OE plants are labelled OE-wtBjHMGS1 and OE-S359A. Two independent lines of OE-wtBjHMGS1 plants, “401” (upper) and “402” (lower) and two independent lines of OE-S359A plants, “603” (upper) and “606” (lower) are shown. Bar  = 10 cm. (E) Statistical analysis on height of 80-d-old transgenic plants. Values are mean ±SD (n = 6); Bars are SD; H, higher than control; a indicates significant difference between HMGS-OE and the vector (pSa13)-transformed control (*P*<0.01 by the Student's *t*-test); b indicates significant difference between OE-wtBjHMGS1 and OE-S359A (*P*<0.01 by the Student's *t*-test). pSa13, vector-transformed control; two independent lines of OE-wtBjHMGS1 (“401” and “402”) and two independent lines of OE-S359A (“603” and “606”) were used for growth rate measurement.

As sterols or steroid plant hormones have been reported to regulate plant growth [8], [56], phenotyping was carried out on 14-d-old seedlings and 80-d-old plants. In 14-d-old HMGS-OE (OE-wtBjHMGS1 and OE-S359A) seedlings, root length (Figure 3A–B) and dry weight (Figure 3C) were significantly greater than the vector (pSa13)-transformed controls. Although the root length of 14-d-old seedlings in OE-S359A was not significantly greater than the OE-wtBjHMGS1 (Figure 3B), their dry weight was significantly heavier than OE-wtBjHMGS1 (Figure 3C). Consistently, 80-d-old tobacco HMGS-OE greenhouse plants grew better than the vector-transformed control (Figure 3D). HMGS-OEs (OE-wtBjHMGS1 and OE-S359A) were taller at 80-d than the control (Figure 3E). More interestingly, 80-d-old OE-S359A displayed significantly greater height than the OE-wtBjHMGS1 (Figure 3E).

**Figure 4:**
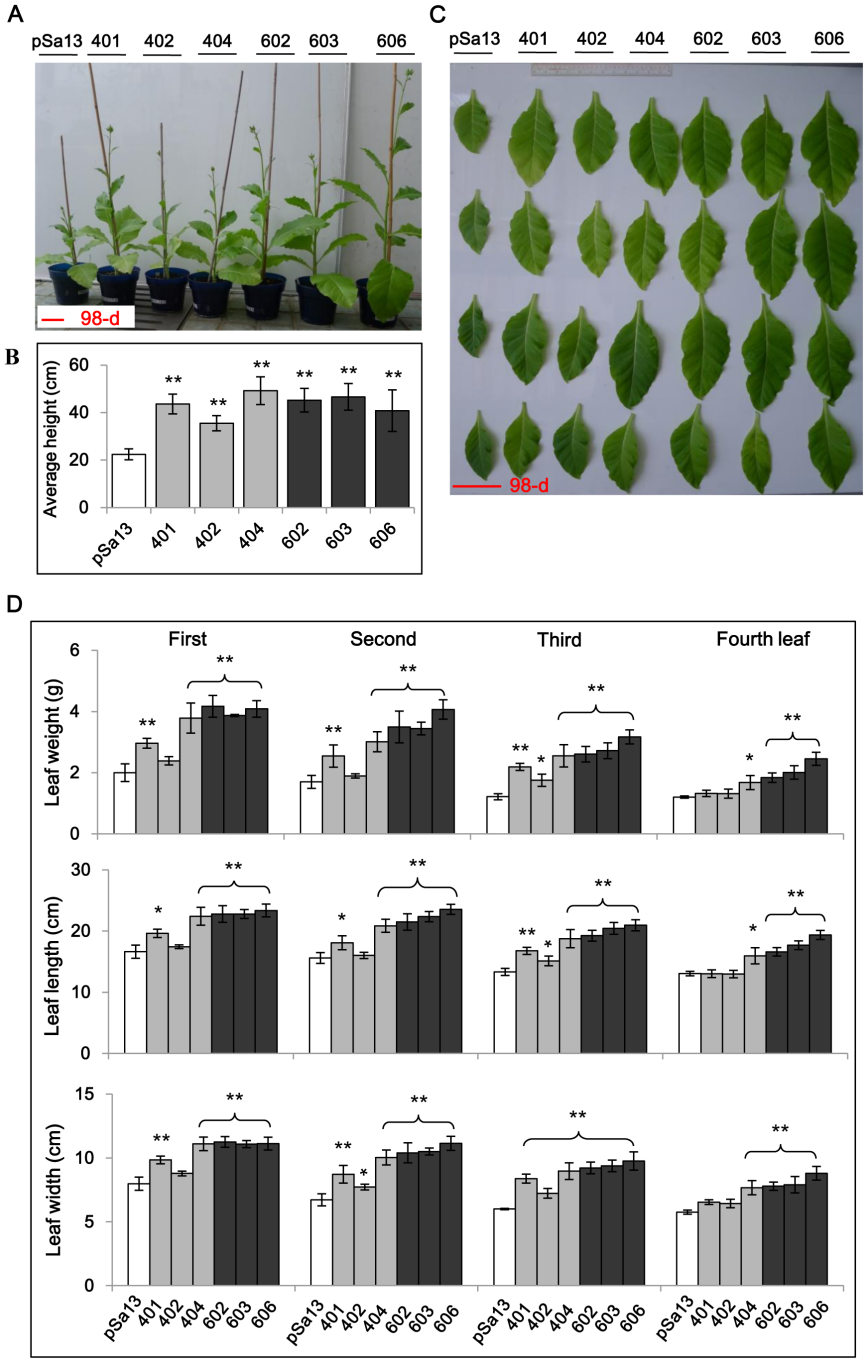
Comparison in plant growth between 98-d-old greenhouse-grown HMGS-OEs and vector-transformed tobacco. (A) Representative plants photographed 98-d after germination show differences in growth between HMGS-OE tobacco plants and vector-transformed control. Bar  = 10 cm. (B) Analysis on height of 98-d-old transgenic plants. (C) Representative tobacco leaves photographed 98-d after germination with growth differences between HMGS-OE and vector-transformed tobacco. Bar  = 10 cm. (D) Analysis on fresh weight, length and width of bottom-most four leaves from a 98-d-old tobacco plant. Values are mean ± SD (n = 6); Bars are SD; **, *P*<0.01; *, *P*<0.05; ** and *, significantly higher than control, by the Student's *t*-test. The vector-transformed control is labelled “pSa13”, three independent lines of OE-wtBjHMGS1 plants are labelled “401”, “402” and “404”, and three independent lines of OE-S359A plants are labelled “602”, “603” and “606”.

Growth differences in height (Figure 4A–B) and leaf size (Figure 4C–D) between 98-d-old HMGS-OEs (OE-wtBjHMGS1 and OE-S359A) and vector (pSa13)-transformed plants were also evident (Figure 4). Both OE-wtBjHMGS1 and OE-S359A had a significant increase (91% and 97%, respectively) in height over the vector-transformed control (Figure 4B). Leaf fresh weight and size (length and width) (Figure 4C–D) in some of the OE-wtBjHMGS1 lines and all three OE-S359A lines were significantly heavier and bigger, respectively, than the control at similar age (Figure 4D).

**Figure 5:**
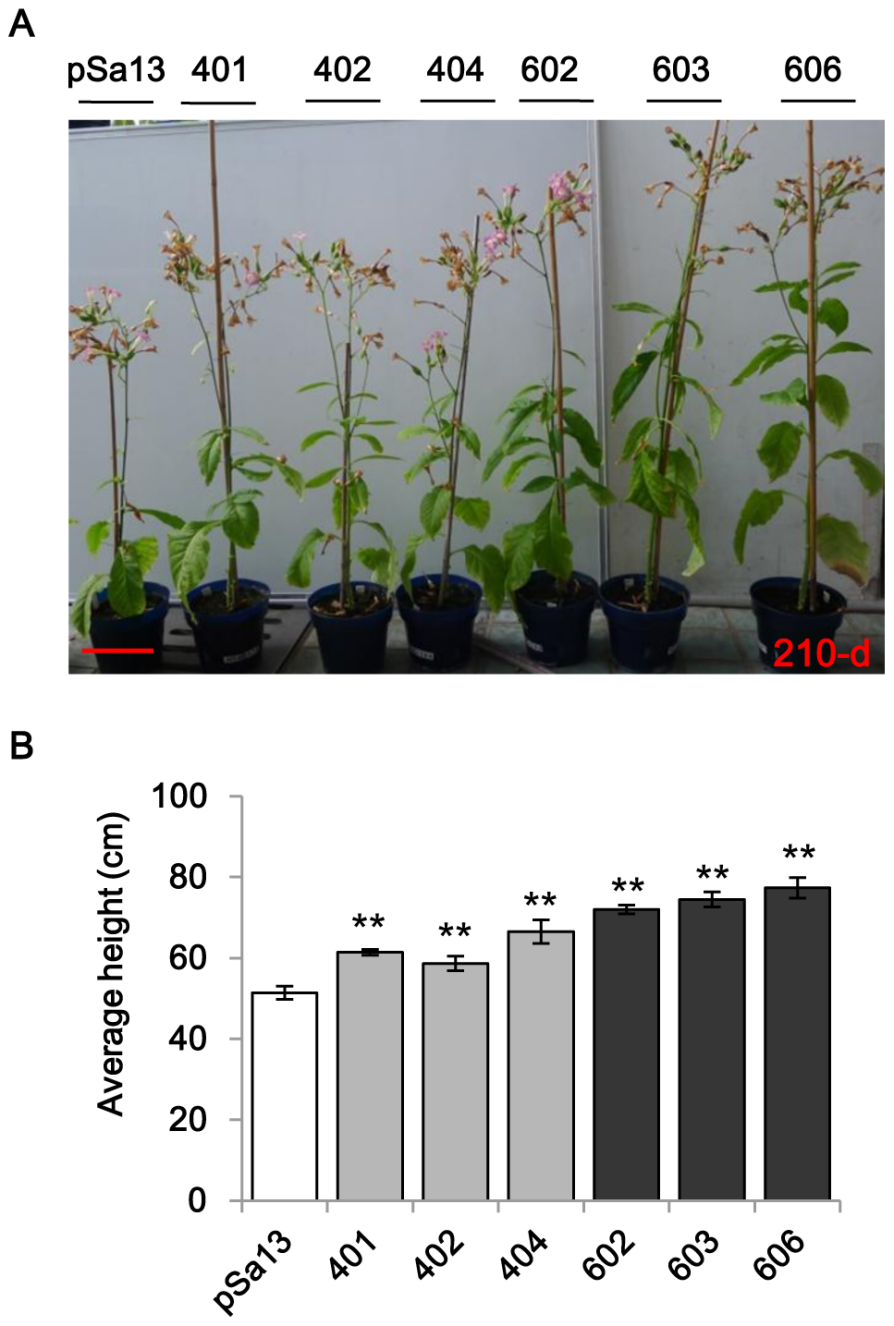
Comparison in plant growth between 210-d-old greenhouse-grown HMGS-OEs and vector-transformed tobacco. (A) Representative plants photographed 210-d after germination show differences in growth between HMGS-OE tobacco plants and the vector (pSa13)-transformed control. Bar  = 10 cm. (B) Analysis on height of 210-d-old transgenic plants. Values are mean ± SD (n = 6); Bars are SD; **, *P*<0.01; *, *P*<0.05; ** and *, significantly higher than control, by the Student's *t*-test. The vector-transformed control is labelled “pSa13”, three independent lines of OE-wtBjHMGS1 plants are labelled “401”, “402” and “404”, and three independent lines of OE-S359A plants are labelled “602”, “603” and “606”.

Furthermore, growth differences in height between 210-d-old HMGS-OEs (OE-wtBjHMGS1 and OE-S359A) and vector-transformed plants were also observed (Figure 5). OE-wtBjHMGS1 showed a significant increase (21%) in height over the control, while OE-S359A displayed an even higher increase (45%) (Figure 5B).

### Tobacco HMGS-OEs produce an enhanced seed yield

**Figure 6:**
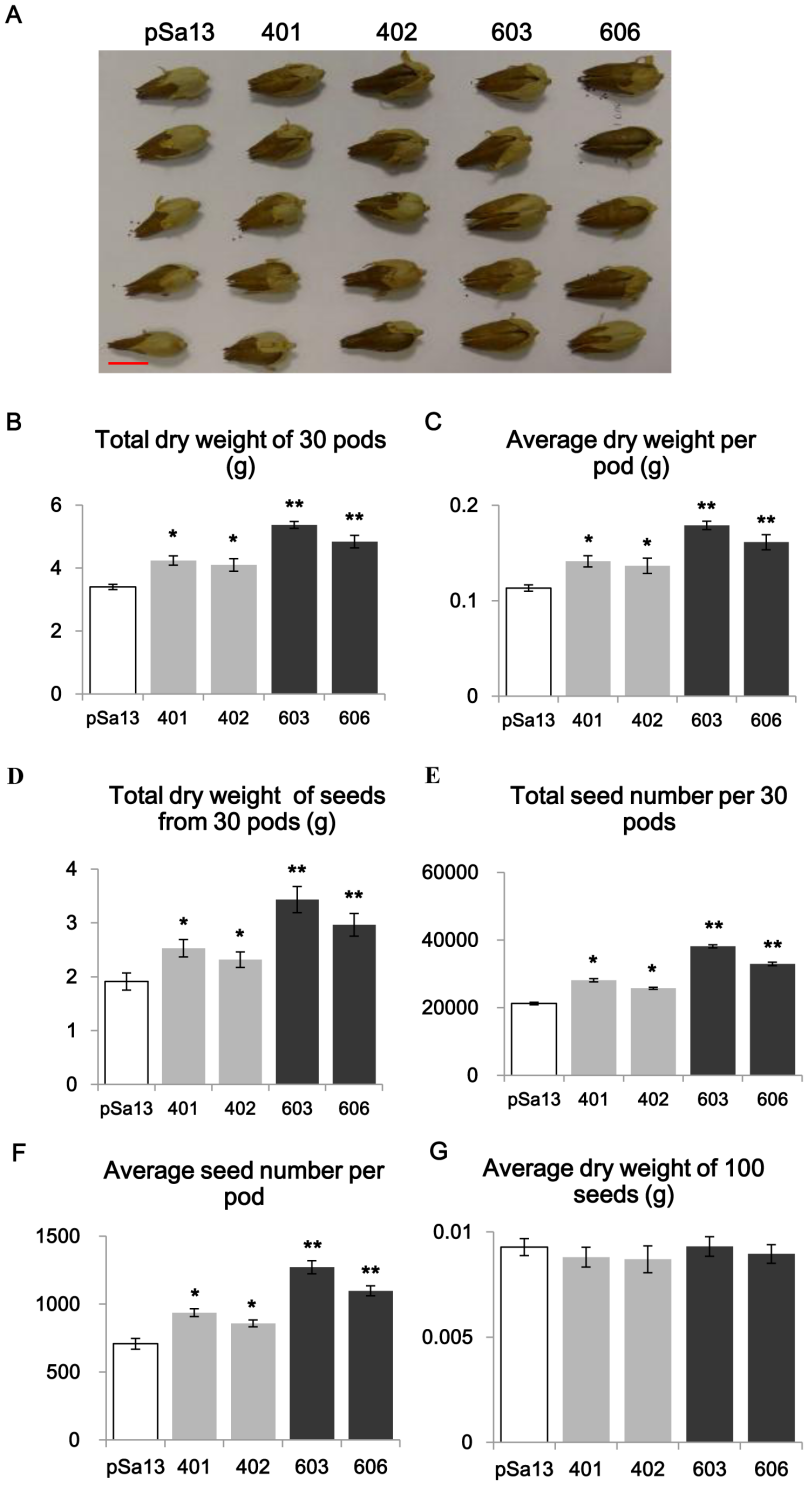
Tobacco HMGS-OEs show increased seed yield. (A) Phenotype of tobacco pods. pSa13, vector-transformed control; “401” and “402”, two independent lines of OE-wtBjHMGS1 and “603” and “606”, two independent lines of OE-S359A. Scale bar  = 1 cm. (B) Total dry weight of 30 tobacco pods. (C) Average dry weight per pod. (D) Total dry weight of seeds from 30 pods. (E) Total seed number per 30 pods. (F) Average seed number per pod. (G) Average dry weight of 100 seeds in control and HMGS-OEs. Thirty independent readings were taken for each line. Values are means ± SD, n = 30. a indicates significant difference between HMGS-OE and the vector (pSa13)-transformed control; b indicates significant difference between OE-wtBjHMGS1 and OE-S359A. H, value higher than the control (P<0.05 or 0.01 by the Student's *t*-test).

Comparison in seed yield by seed weight measurement between HMGS-OEs (OE-wtBjHMGS1 and OE-S359A) and the vector (pSa13)-transformed control indicated that both OE-wtBjHMGS1 and OE-S359A were higher than the control (Figure 6A–D); seed yield of OE-wtBjHMGS1 increased by 21 to 32% (*P*<0.05) (Figure 6D–F), while OE-S359A showed a 55 to 80% rise (*P*<0.01) (Figure 6D–F). OE-S359A (lines “603” and “606”) showed an average of 32% increase over OE-wtBjHMGS1 (lines “401” and “402”) by the Student's *t*-test (*P*<0.05) (Figure 6D–F). No significant difference in dry seed weight of 100 seeds was noted between the vector-transformed control and HMGS-OEs (Figure 6G), suggesting that seed size was not affected. Hence, HMGS-OE increase in seed yield was attributed to increase in pod size and seed number rather than seed size (Figure 6).

### Change in expression of isoprenoid biosynthesis genes in tobacco HMGS-OEs

**Figure 7:**
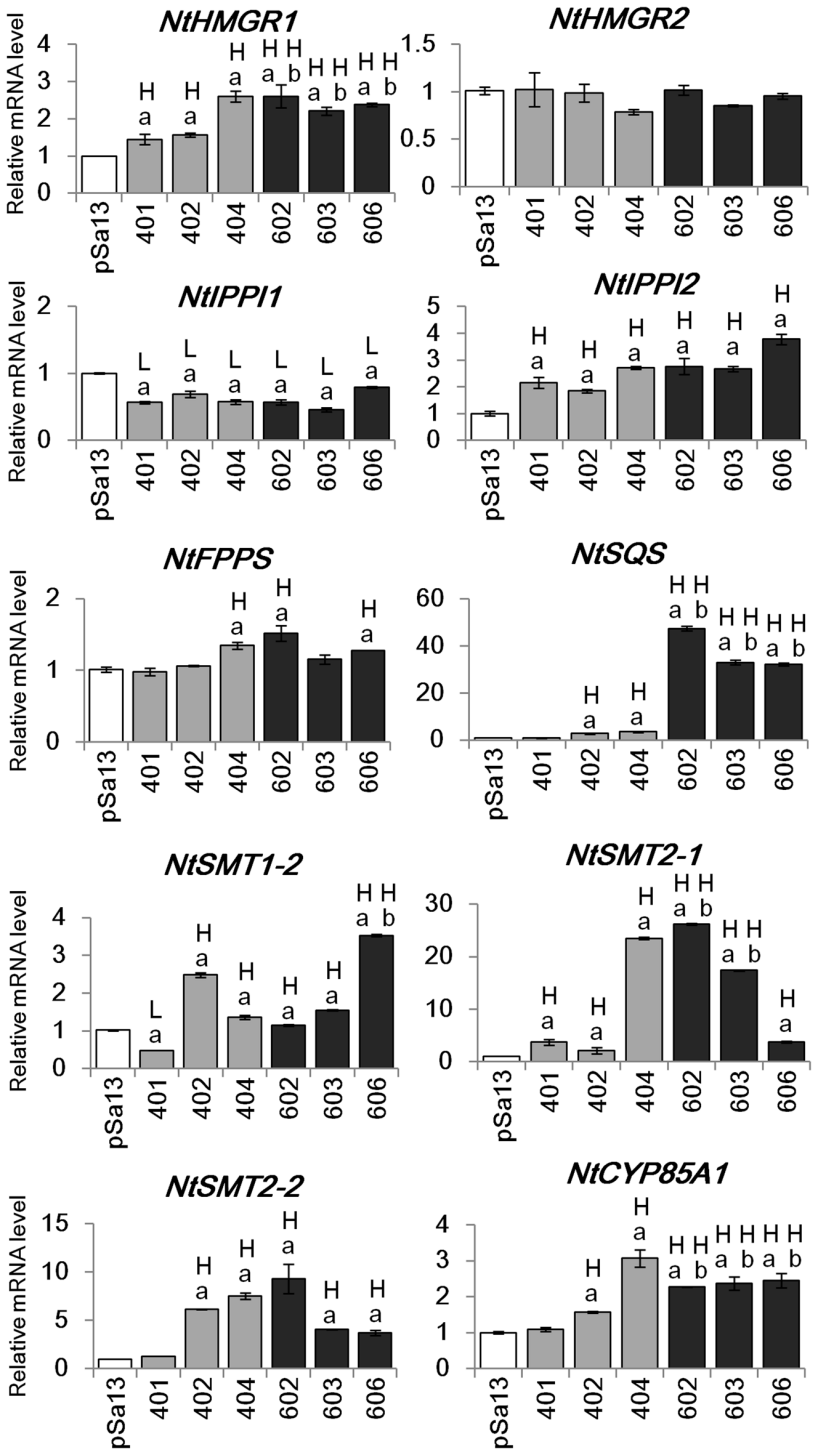
Expression of HMGS downstream genes by qRT-PCR in 20-d-old tobacco seedlings of HMGS-OEs. Total RNA was extracted from 20-d-old tobacco seedlings of vector (pSa13)-transformed control, three independent lines of OE-wtBjHMGS1 (lines “401”, “402” and “404”) and three independent lines of OE-S359A (lines “602”, “603” and “606”). H, value higher than the control (*P*<0.05, Student's *t*-test); L, value lower than the control (*P*<0.05, Student's *t*-test). Values are means ±SD (n = 3). a indicates significant difference between HMGS-OE and the vector (pSa13)-transformed control for at least two independent lines from three independent lines; b indicates significant difference between OE-wtBjHMGS1 and OE-S359A for at least two independent lines from three independent lines.

**Figure 8:**
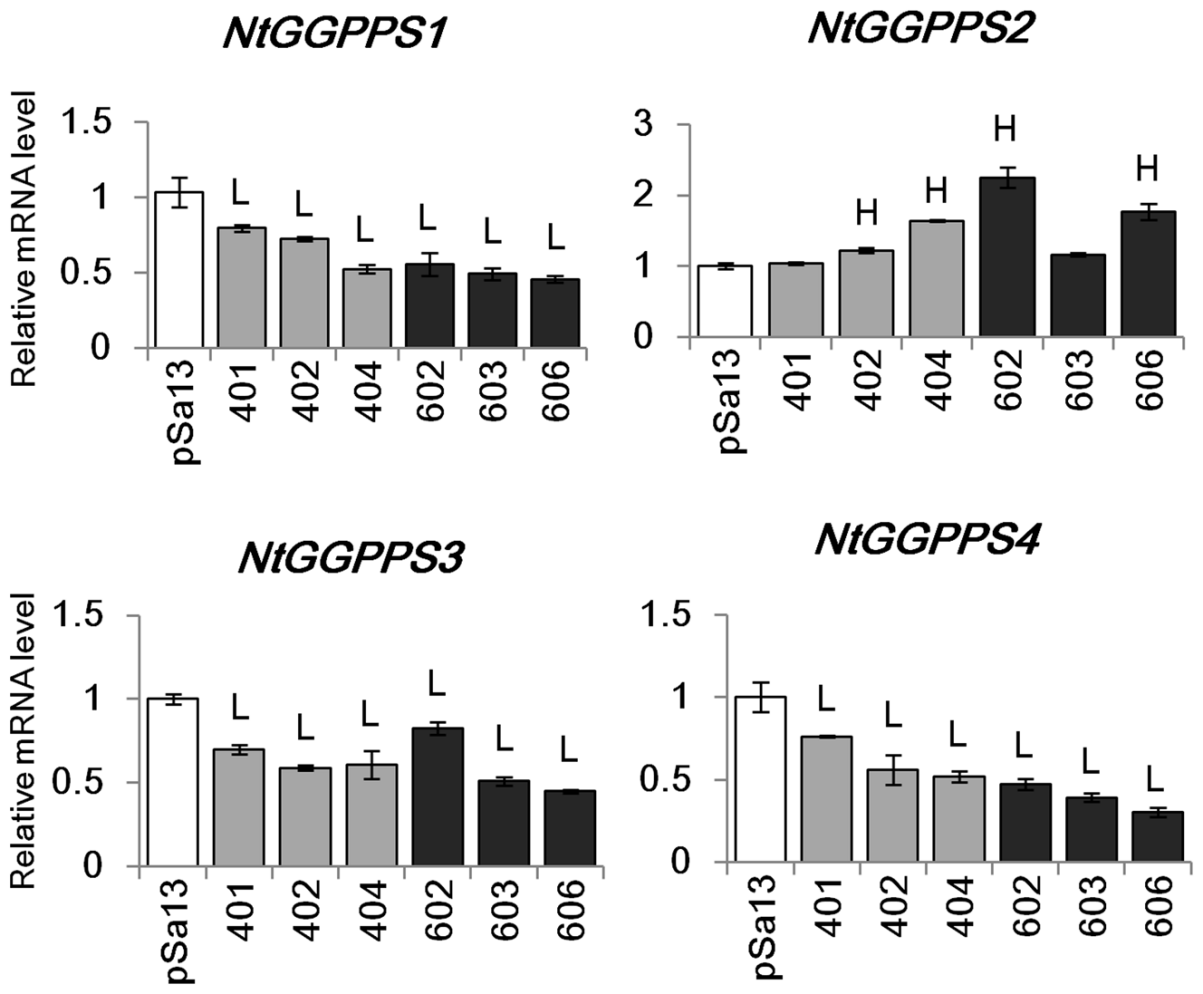
Expression of plastidial *GGPPSs* determined by qRT-PCR in 20-d-old tobacco seedlings of HMGS-OEs. Total RNA was extracted from 20-d-old tobacco seedlings of vector (pSa13)-transformed control, three independent lines of OE-wtBjHMGS1 (lines “401”, “402” and “404”) and three independent lines of OE-S359A (lines “602”, “603” and “606”). H, value higher than the control (*P*<0.05, Student's *t*-test); L, value lower than the control (*P*<0.05, Student's *t*-test). Values are means ± SD (n = 3).

qRT-PCR was performed to check the effect of *BjHMGS1* overexpression on the expression of genes downstream of *HMGS* in tobacco HMGS-OE seedlings and to explore possible molecular mechanism of HMGS function in plant growth and seed production. The results from qRT-PCR revealed that the expression of *NtHMGR1*, *NtIPPI2*, *NtSQS*, *NtSMT1-2*, *NtSMT2-1*, *NtSMT2-2* and *NtCYP85A1* was significantly higher than in the vector (pSa13)-transformed control for both OE-wtBjHMGS1 and OE-S359A tobacco seedlings with the exception of *NtSQS*, *NtSMT1-2*, *NtSMT2-2* and *NtCYP85A1* in one OE-wtBjHMGS1 line (401) (*P*<0.01) (Figure 7). However, there was no difference in the expression of *NtHMGR2* between all the HMGS-OE lines and the vector-transformed control (Figure 7). For the expression of *NtFPPS*, there was no disparity amongst the two lines of OE-wtBjHMGS1 (401 and 402) and the vector-transformed control, while the expression of *NtFPPS* in another OE-wtBjHMGS1 line (404) and in two OE-S359A lines (602 and 606) was slightly higher than the control (*P*<0.05) (Figure 7). Conversely, the expression of *NtIPPI1*, *NtGGPPS1*, *NtGGPPS3* and *NtGGPPS4* were down-regulated in tobacco HMGS-OE seedlings (*P*<0.01) (Figures 7–8) while the expression of *NtGGPPS2* was higher than the control (*P*<0.05) in two OE-wtBjHMGS1 lines (402 and 404) and two OE-S359A lines (602 and 606) (Figure 8). Observations that (i) *NtSQS* expression in all three OE-S359A lines was higher than all three OE-wtBjHMGS1 lines, (ii) *NtHMGR1* and *NtCYP85A1* expression in all three OE-S359A lines were higher than two (“401” and “402”) of three OE-wtBjHMGS1 lines, and (iii) *NtSMT2-1* expression in two (“602” and “603”) of three OE-S359A lines was higher than two (“401” and “402”) of three OE-wtBjHMGS1 lines suggest that the differences in expression levels of *NtSQS*, *NtHMGR1*, *NtSMT2-1* and *NtCYP85A1* in OE-wtBjHMGS1 and OE-S359A do correspond to the expected differences in enzyme activities between recombinant wtBjHMGS1 and S359A [37].

## Discussion

### New observations from tobacco HMGS-OEs

Our investigations on the overexpression of HMGS in transgenic tobacco revealed new observations not previously evident in *Arabidopsis* HMGS-OEs including the upregulation of *NtIPPI2*, *NtSQS* and *NtGGPPS2* and downregulation of *NtIPPI1*, *NtGGPPS1*, *NtGGPPS3* and *NtGGPPS4* (Figures 7–8). However, similar to findings from *Arabidopsis* HMGS-OEs, enhanced *NtHMGR1*, *NtSMT1-2*, *NtSMT2-1*, *NtSMT2-2* and *NtCYP85A1* expression in tobacco HMGS-OEs was seen (Figure 7). Other new findings from tobacco HMGS-OEs included growth stimulation in the tobacco HMGS-OE lines, confirming the positive role of HMGS overexpression in plant growth. Furthermore, tobacco HMGS-OEs show increased pod size and seed yield (Figure 6), indicative of a specific HMGS function in seed production. Improved growth, pod size and seed yield of OE-S359A in comparison to OE-wtBjHNMGS1 may be attributed to the higher *NtSQS* expression (Figure 7) and sterol content in OE-S359A transformants (Table 1).

### Function of HMGS in reproduction and development

In plants, the floral organs are involved in reproduction. HMGS has been shown to play a crucial role in floral development [4], [34], [35], [37]. In *Arabidopsis*, higher *AtHMGS* expression had been observed in flowers than seedlings or leaves from RT-PCR analysis [4]. Using mutants in *HMGS*, *AtHMGS* was demonstrated essential for pollen fertility and proper development of tapetum-specific organelles in *Arabidopsis* [35]. In *B. juncea*, northern blot analysis had previously revealed that *BjHMGS1* mRNA was highly expressed in flowers and seedling hypocotyls [34] and *in situ* hybridization analysis had shown that *HMGS* mRNA was predominantly localized in the stigmata and ovules of flower buds and in the piths of seedling hypocotyls [37]. *BjHMGS1* and *BjHMGS2*, but not *BjHMGS3* and *BjHMGS4* expression was detected in the floral buds as examined by RT-PCR analysis [37]. The effect on the overexpression of BjHMGS1 in transgenic tobacco observed herein further extends the significance of HMGS in reproduction related to seed production as well as to whole plant development (Figures 3–6). More interestingly, OE-S359A lines were found to display greater effect in growth, pod size and seed yield than OE-wtBjHNMGS1 (Figures 3–6). OE-S359A, which was expected to possess higher HMGS activity than OE-wtBjHMGS1, caused higher expression of tobacco native genes downstream of HMGS such as *NtSQS*, *NtHMGR1*, *NtSMT2-1* and *NtCYP85A1* (Figure 7), and increased sterol levels, which more effectively enhanced seed production in comparison to OE- wtBjHMGS1.

Besides HMGS, other enzymes in the early steps of the MVA pathway are important in these development processes. It has been observed that both *hmg1/hmg1* and *HMG1/hmg1 hmg2/hmg2 Arabidopsis* mutants deficient in HMGR activity are male sterile [26], [57]. The *hmg1hmg2* male gametophytes in the *HMG1/hmg1 hmg2/hmg2* mutant were lethal [57]. Furthermore, the characterization of *Arabidopsis AACT1* and *AACT2* led to suggest a specific role of AACT2 in catalyzing the first step of the MVA pathway [58], while AACT1 is rather involved in the peroxisomal fatty acid degradation process, like in tobacco seedlings [59]. *Arabidopsis AACT2 RNAi* lines further showed reduction in apical dominance, seed yield and root length accompanied by sterility and dwarfing [25]. These studies using the *AACT* RNAi lines, and mutants in *HMGS* and *HMGR* together with observations herein confirm the significance of the MVA pathway in plant reproduction and development.

Recently, two genes were cloned and characterized from two miRNA-action deficient (*MAD*) mutants; *MAD3* encodes the MVA pathway enzyme HMGR1, while *MAD4* encodes sterol C-8 isomerase in dedicated sterol biosynthesis [60]. Their results showed that the lack in HMGR1 catalytic activity is sufficient to inhibit miRNA activity and that sterol is essential for the normal activity of plant miRNAs [60]. Furthermore, their results implied that besides sterols, other isoprenoids may also affect the normal function of miRNA [60]. It has been reported that *Caenorhabditis elegans* HMGS1 (CeHMGS1) plays an important role in the miRNA pathway; *CeHMGS1* regulates the function of many, if not all, miRNAs at multiple tissues and stages during *C. elegans* development [61]. Furthermore, *CeHMGS1* affects the fertility of *C. elegans* in the miRNA defective *let-7* worms [61]. This effect on fertility is reminiscent of our observations on tobacco HMGS-OEs herein on seed production which represents fertility in plants.

### Effects of HMGS in regulating isoprenoid biosynthesis genes in tobacco HMGS-OEs

In transgenic *Arabidopsis*, the overexpression of wt and mutant (H188N, S359A and H188N/S359A) BjHMGS1 caused a feed-forward effect in the upregulation of several genes in sterol biosynthesis including *HMGR*, *SMT2*, *DWF1*, *CYP710A1* and *BR6OX2* [4]. This study using tobacco HMGS-OEs demonstrated that some differences exist between tobacco and *Arabidopsis* HMGS-OEs in the expression of genes encoding HMGR and SMT (cf. Figure 1). Although HMGR is considered to be the rate-limiting enzyme in the MVA pathway in plants [62], only *NtHMGR1* but not *NtHMGR2* was upregulated in tobacco HMGS-OEs (Figure 7). This can perhaps be attributed to some differences in the localization and function of NtHMGR1 and NtHMGR2 [63], [64]. *NtHMGR1* is a house-keeping gene that likely participates in sterol biosynthesis, plant growth and development, while *NtHMGR2* is stress-inducible [63], [64]. Also elicitor-inducible HMGR activity is known to be associated with defence-related sesquiterpenoid accumulation in tobacco cell suspension cultures [65]. Thus it was not surprising that rather than *NtHMGR2*, *NtHMGR1* was upregulated in seedlings undergoing rapid growth and development.

Isopentenyl diphosphate isomerase (IPPI) catalyses the interconversion of IPP and its allyl isomer dimethylallyl diphosphate (DMAPP) and provides the first key intermediate for the biosynthesis of all kinds of isoprenoids including sterols in the MVA pathway and carotenoids in the MEP pathway [1], [3], [12], [66] (and references cited therein) (cf. Figure 1). IPP is most likely involved in cross-talk between the cytosolic MVA pathway and the plastidial MEP pathway [13], [14]. *AtIPPI1* and *AtIPPI2* have been reported to be critical to sterol biosynthesis in the MVA pathway and *Arabidopsis* development [67]. Analysis of the expression of the two *NtIPPI* genes in tobacco HMGS-OE seedlings revealed that *NtIPPI1* was downregulated, while *NtIPPI2* was upregulated (Figure 7). Their corresponding proteins are apparently differentially localized in tobacco [68]. NtIPPI1 is targeted to the chloroplast, while NtIPPI2 is cytosolic, similar to BjHMGS1 [37], [68]. Possibly, upregulation of *BjHMGS1* and *NtIPPI2* in the cytosol of tobacco HMGS-OE seedlings promoted cross-talk between the MVA and MEP pathways. The MEP pathway produces simultaneously IPP and DMAPP, and plastidial NtIPPI1 is possibly needed to adjust the ratio of starter DMAPP to elongation units IPP for longer prenyl chains. If IPP is imported from the cytosol because of “overproduction”, then plastidial *NtIPPI1* would be downregulated.

FPPS catalyses the condensation of two molecules of IPP with DMAPP to form farnesyl diphosphate (FPP) (C_15_) (cf. Figure 1), which provides the key precursor for the biosynthesis of essential isoprenoids such as sesquiterpenes, ubiquinones, polyterpenes, dolichols and sterols [69], [70]. In plants, FPPS isozymes that are encoded by a small gene family, exert differential roles, based on their subcellular localisation [69], [71]. *NtFPPS* expression was slightly elevated in seedlings of only one OE-wtBjHMGS1 line (Figure 7). Given that NtFPPS functions as the key provider of the universal product FPP in the biosynthesis of many C-15 related products, a moderate change in *NtFPPS* mRNA in the HMGS-OE lines may not be significant enough to affect sterol accumulation. Also, other *NtFPPS* isogenes or post-translational regulation may be involved [72]–[74].

SQS catalyses the biosynthesis of squalene by the reductive dimerization of two FPP molecules (cf. Figure 1), and represents the first committed step in the biosynthesis of sterols, BRs and triterpenes [75]–[79]. The change in *NtSQS* expression in seedlings was the most dramatic, with a 2.1-fold increase in two lines of OE-wtBjHMGS1 and 36.5-fold in OE-S359A, in comparison to the vector-transformed control (Figure 7). The increase of *NtSQS* mRNA in OE-S359A seedlings was also much higher (11.1-fold) than OE-wtBjHMGS1 (Figure 7). Interestingly, *NtSQS* expression and NtSQS activity have been detected predominantly at the shoot apical meristem (SAM) rather than leaves or roots, implying that sterol biosynthesis occurs especially in the SAM [77]. Furthermore, the SAM is critical in plant growth and development, and stem cells from the SAM continuously generate all the aerial organs and tissues of a plant [80]. Results from qRT-PCR (Figure 7) herein support a role for *NtSQS* in *HMGS*-associated sterol accumulation related to growth and seed yield. Also, enhanced sterol accumulation, growth and seed yield in OE-S359A, over OE-wtBjHMGS1 (Figure 7), corresponded to higher *NtSQS* expression (Figure 7). Consistently, Arabidopsis *SQS* (*AtSQS*) displayed higher expression in HMGS-OEs than the vector-transformed control; and *AtSQS* expression in OE-S359A was higher than OE-wtBjHMGS1 (Figure S4). However the elevation of *NtSQS* in tobacco OE-S359A over OE-wtBjHMGS1 (Figure 7) was greater in comparison to *AtSQS* in Arabidopsis OE-S359A (Figure S4). Furthermore, our results correspond well to a recent study on the overexpression of *Glycine max* SQS1 (GmSQS1) in Arabidopsis that yielded a 50% increase of seed sterol content [81]. An enhanced flux of MVA to FPP might present some risk as phosphatases always being present might liberate farnesol, which can be quite toxic to cells [82]. Thus SQS could remove a potentially dangerous intermediate and get it channelled into the synthesis and accumulation of chemically inert sterols and their derivatives.

In the MEP pathway, GGPPS catalyses the consecutive condensation of three molecules of IPP and one DMAPP to generate the 20-carbon geranylgeranyl diphosphate (GGPP) (cf. Figure 1), which is the universal key intermediate for the biosynthesis of carotenoids and of abscisic acid as derivative, of gibberellins, chlorophylls, tocopherols, phylloquinone, plastoquinone, dolichols, polyprenols and oligoprenols [12], [83]. Although four GGPPS-like cDNAs have been reported from tobacco [84], only *NtGGPPS2* was upregulated in two lines of OE-wtBjHMGS1 and all three lines of OE-S359A, while *NtGGPPS1*, *NtGGPPS3* and *NtGGPPS4* were observed to be downregulated in all the HMGS-OE seedlings (Figure 8), implying that HMGS overexpression had a positive effect on *NtGGPPS2* expression and a negative role on *NtGGPPS1*, *NtGGPPS3* and *NtGGPPS4* expression. However, it cannot be discounted that NtGGPPS1, NtGGPPS3 and NtGGPPS4 may be subject to other modes of regulation such as post-translational modification that has been reported for AtGGPPS3, AtGGPPS7, AtGGPPS9 and AtGGPPS10 [85], [86]. Most recently, a new relationship between the MVA pathway and the MEP pathway has been proposed in which the monoterpene *S*-carvone inhibited the production of MVA-derived capsidiol, a cellulose-induced sesquiterpenoid phytoalexin in tobacco by down-regulation of MEP-pathway dependent protein isoprenylation [87].

The overexpression of NtSMT1(cf. Figure 1), which catalyses the conversion of cycloartenol to 24-methylene cycloartanol, considered as the first methylation step in phytosterol biosynthesis, resulted in a higher total sterol content in tobacco seeds [88]–[90]. Transgenic tobacco overexpressing AtSMT2/NtSMT2, which converts 24-methylene lophenol to 24-ethylidene lophenol, showed an increase in sitosterol but not total sterol content [91]–[94]. HMGS overexpression in tobacco upregulated both *NtSMT1* and *NtSMT2* expression in seedlings of all three OE-S359A lines and two OE-wtBjHMGS1 lines with the exception of OE-wtBjHMGS1 line 401 (Figure 7). The upregulation of *SMT2* was also observed in 21-d-old rosette leaves of transgenic *Arabidopsis* overexpressing *BjHMGS1* [4]. Our results suggest that *NtSMT1* affects *HMGS*-associated sterol accumulation, which had not been previously observed in transgenic *Arabidopsis* HMGS-OEs [4].

BR is a steroid hormone essential for plant growth and development [95]. Several mutants in BR biosynthesis affect seed yield [6], [22]–[24]. The cytochrome P-450 monooxygenases (CYP85A family) are involved in the last several oxidative reactions in the BR pathway [96]. In *Arabidopsis*, two members of CYP85A exist: AtCYP85A1 (brassinosteroid-6-oxidase 1, BR60X1) that catalyses several reactions in the biosynthesis of castasterone [97], and AtCYP85A2 (BR60X*2*) in the conversion of castasterone to brassinolide [96]. The *Arabidopsis cyp85a1* mutant showed a semi-sterile phenotype and the *cyp85a2* mutant exhibited dwarfness and reduced fertility [96]–[97]. The *cyp85a1/cyp85a2* double mutants displayed severe dwarfism [96]. To test the effect in HMGS overexpression on BR biosynthesis, *NtCYP85A1* (cf. Figure 1) mRNA was measured in tobacco HMGS-OE seedlings and was observed to significantly increase in all three OE-S359A lines and two OE-wtBjHMGS1 lines with the exception of OE-wtBjHMGS1 line 401 (Figure 7). Although OE-wtBjHMGS1 line 401 did not show higher expression in *NtCYP85A1*, as well as in *NtSQS*, *NtSMT1-2*, *NtSMT2-2* and *NtGGPPS2*, the expression of all these genes were maintained a level similar to the control (Figures 7–8). Furthermore, *NtHMGR1*, *NtIPPI2* and *NtSMT2-1* displayed significantly higher expression in this line than the control (Figure 7), implying that they positively affected plant growth and seed yield. Taken together with observations on a general up-regulation of *AtCYP85A2* (*BR60X2*) in 21-d-old rosette leaves of transgenic *Arabidopsis* overexpressing BjHMGS1 [4], our studies reinforce that HMGS overexpression likely leads to upregulation of BR synthesis, and thereby promotes growth and seed production.

## Supporting Information

Figure S1 **The BjHMGS1 constructs used in tobacco transformation and resultant PCR analysis on transgenic tobacco lines.** (A) Schematic map of transformation vector indicating primer location. *BjHMGS1* wild-type and mutant inserts were derived from plasmids, pBj134 (WT *BjHMGS1*) and pBj136 (S359A) [4]. *CaMV35S*: Cauliflower Mosaic Virus *35S* promoter; *NOSpro*: nopaline synthase (*NOS*) promoter; *NOSter*: *NOS* terminator; *NPTII*: gene encoding neomycin phosphotransferase II conferring resistance to kanamycin; RB: right border of T-DNA; LB: left border of T-DNA. *35S*: *35S* promoter 3'-end forward primer; ML264: *BjHMGS1-*specific 3'-end reverse primer. (B) Agarose gel showing the expected 1.65-kb *BjHMGS1* cDNA band (arrowed) from transgenic tobacco following PCR using primer pair 35S/ML264; representative lines are shown here. OE-wtBjHMGS1 (lanes 1–3); OE-S359A (lanes 4–6); positive control (PC) (lane 7, PCR template plasmid pBj134); blank control (BC) (lane 8, no DNA band after PCR). Putative tobacco HMGS-OEs were designated as OE-wtBjHMGS1 (lines “401”, “402” and “404”) and OE-S359A (lines “602”, “603” and “606”).
(TIF)

Figure S2 **Southern blot analysis on transgenic tobacco plants.** (A) Schematic map of transformation vector indicating *Eco*RI (E) sites. *BjHMGS1* wild-type and mutant inserts were derived from plasmids pBj134 (wt*BjHMGS1*) and pBj136 (S359A). *CaMV35S*: Cauliflower Mosaic Virus *35S* promoter; *NOSpro*: nopaline synthase (*NOS*) promoter; *NOSter*: *NOS* terminator; *NPTII*: gene encoding neomycin phosphotransferase II conferring resistance to kanamycin; RB: right border of T-DNA; LB: left border of T-DNA. Dotted lines denote position of nucleotide on vector. (B) Southern blot analysis of genomic DNA digested by restrictive endonuclease *Eco*RI and probed with ^32^P-labelled *BjHMGS1* full-length cDNA in representative blots. Arrowheads indicate hybridizing bands. OE-wtBjHMGS1 transformants (lanes 1–2), OE-S359A transformants (lanes 3–5). Representative single insertion lines (transformants “401” and “402” for OE-wtBjHMGS1 and “603” and “606” for OE-S359A) are underlined. Transformant “601” likely has a more than one inserts and was not included in further analysis.
(TIF)

Figure S3 **Comparison in seed germination of tobacco HMGS-OEs.** Statistical data on seed germination rates recorded at 60, 72, 84, 96, 108 and 120 h after incubation at 23°C indicates (a) significant difference (P<0.01 by the Student's *t*-test) between HMGS-OE and the vector (pSa13)-transformed control; (b) indicates significant difference (P<0.01 by the Student's *t*-test) between OE-wtBjHMGS1 and OE-S359A. Values are mean ±SD (n = 5); bars represent SD. pSa13, vector-transformed control; the two independent lines of OE-wtBjHMGS1 (“401” and “402”) and two independent lines of OE-S359A (“603” and “606”) were tested in seed germination assays. The data represents the average from two transformants.
(TIF)

Figure S4 **Expression of Arabidopsis** ***SQS*** **by qRT-PCR in 14-d-old HMGS-OE seedlings.** Total RNA was extracted from 14-d-old Arabidopsis seedlings of vector (pSa13)-transformed control, two independent lines of OE-wtBjHMGS1 (lines “134-L1” and “134-L2”) and two independent lines of OE-S359A (lines “136-L1” and “136-L2”) previously generated [4]. H, value higher than the control (*P*<0.01, Student's *t*-test). Values are means ± SD (n = 3). a indicates significant difference between HMGS-OE and the vector (pSa13)-transformed control; b indicates significant difference between OE-wtBjHMGS1 and OE-S359A.
(TIF)

Table S1 **Oligonucleotide primers used in this study.** Restriction sites are underlined.
(DOCX)

Table S2 **Increase (%) of sterol composition in Arabidopsis HMGS-OE seedlings and leaves in comparison to vector (pSa13)-transformed control.**
(DOCX)
