# Correction: Transgenic tobacco overexpressing *Brassica juncea* HMG-CoA synthase 1 shows increased plant growth, pod size and seed yield

**DOI:** 10.1371/journal.pone.0108026

**Published:** 2014-09-05

**Authors:** The PLOS ONE Staff

There is an error in affiliation 1 for authors Pan Liao, Hui Wang, Mingfu Wang, An-Shan Hsiao, and Mee-Len Chye. Affiliation 1 should be: School of Biological Sciences, The University of Hong Kong, Pokfulam Road, Hong Kong, China.
