# Supplementary figures and images for: Transgenic Tobacco Overexpressing Brassica juncea HMG-CoA Synthase 1 Shows Increased Plant Growth, Pod Size and Seed Yield
Source: PLoS One. 2014 May 21;9(5):e98264. doi: 10.1371/journal.pone.0098264 (PMC4029903; doi:10.1371/journal.pone.0098264)

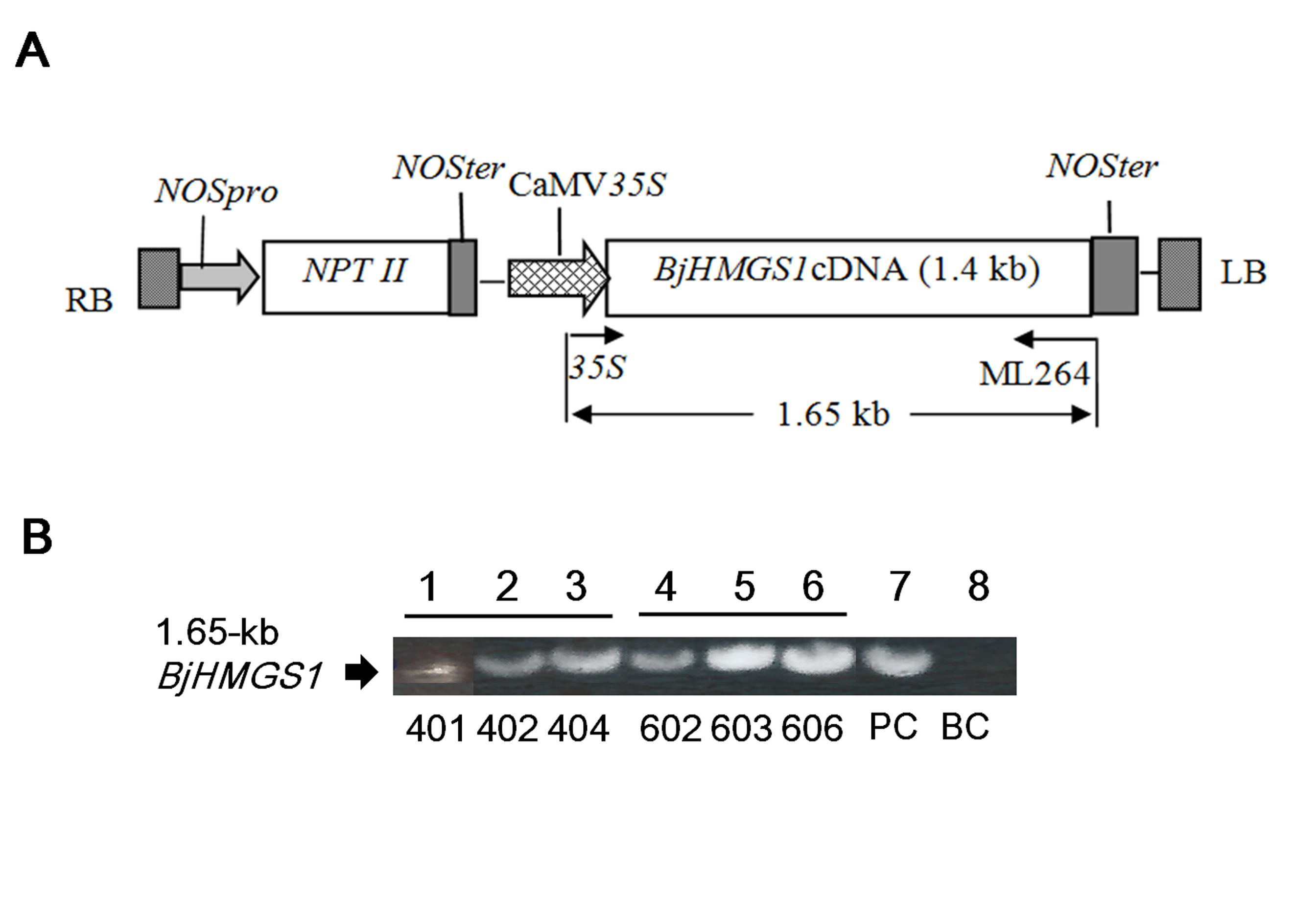

Supplement: Figure S1 — The BjHMGS1 constructs used in tobacco transformation and resultant PCR analysis on transgenic tobacco lines. (A) Schematic map of transformation vector indicating primer location. BjHMGS1 wild-type and mutant inserts were derived from plasmids, pBj134 (WT BjHMGS1) and pBj136 (S359A) [4]. CaMV35S: Cauliflower Mosaic Virus 35S promoter; NOSpro: nopaline synthase (NOS) promoter; NOSter: NOS terminator; NPTII: gene encoding neomycin phosphotransferase II conferring resistance to kanamycin; RB: right border of T-DNA; LB: left border of T-DNA. 35S: 35S promoter 3'-end forward primer; ML264: BjHMGS1-specific 3'-end reverse primer. (B) Agarose gel showing the expected 1.65-kb BjHMGS1 cDNA band (arrowed) from transgenic tobacco following PCR using primer pair 35S/ML264; representative lines are shown here. OE-wtBjHMGS1 (lanes 1–3); OE-S359A (lanes 4–6); positive control (PC) (lane 7, PCR template plasmid pBj134); blank control (BC) (lane 8, no DNA band after PCR). Putative tobacco HMGS-OEs were designated as OE-wtBjHMGS1 (lines “401”, “402” and “404”) and OE-S359A (lines “602”, “603” and “606”). (TIF) [file pone.0098264.s001.tif]

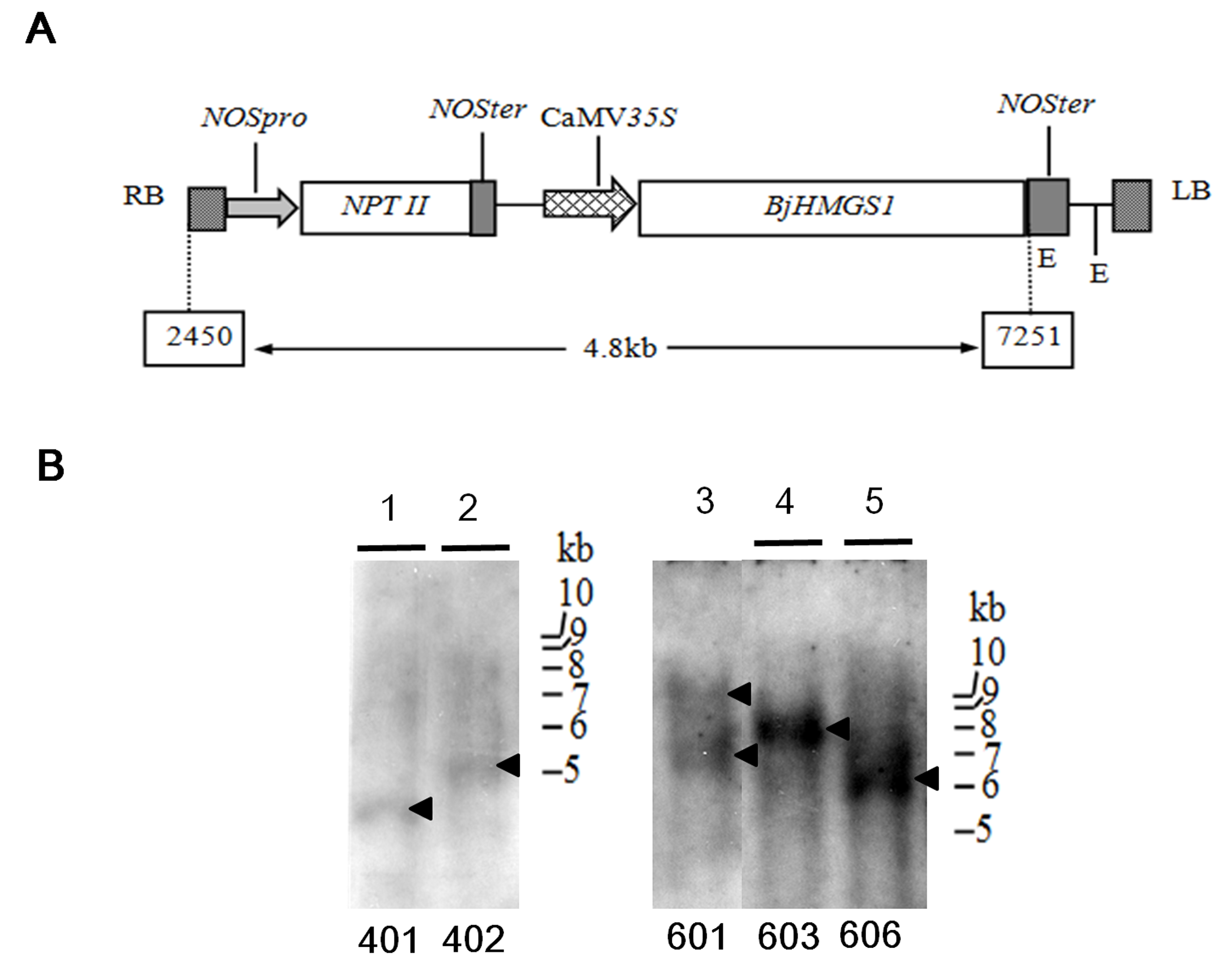

Supplement: Figure S2 — Southern blot analysis on transgenic tobacco plants. (A) Schematic map of transformation vector indicating EcoRI (E) sites. BjHMGS1 wild-type and mutant inserts were derived from plasmids pBj134 (wtBjHMGS1) and pBj136 (S359A). CaMV35S: Cauliflower Mosaic Virus 35S promoter; NOSpro: nopaline synthase (NOS) promoter; NOSter: NOS terminator; NPTII: gene encoding neomycin phosphotransferase II conferring resistance to kanamycin; RB: right border of T-DNA; LB: left border of T-DNA. Dotted lines denote position of nucleotide on vector. (B) Southern blot analysis of genomic DNA digested by restrictive endonuclease EcoRI and probed with 32P-labelled BjHMGS1 full-length cDNA in representative blots. Arrowheads indicate hybridizing bands. OE-wtBjHMGS1 transformants (lanes 1–2), OE-S359A transformants (lanes 3–5). Representative single insertion lines (transformants “401” and “402” for OE-wtBjHMGS1 and “603” and “606” for OE-S359A) are underlined. Transformant “601” likely has a more than one inserts and was not included in further analysis. (TIF) [file pone.0098264.s002.tif]

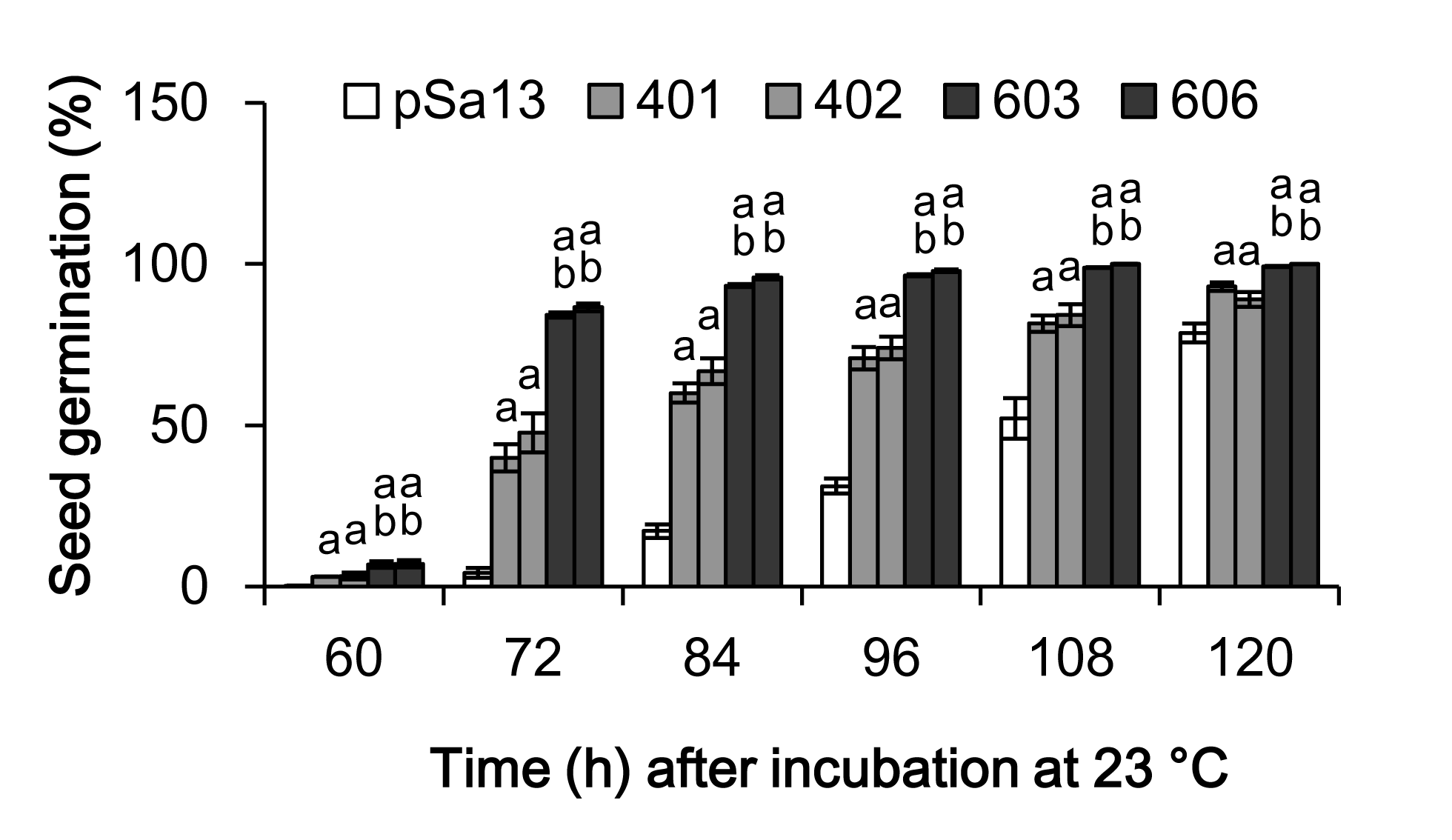

Supplement: Figure S3 — Comparison in seed germination of tobacco HMGS-OEs. Statistical data on seed germination rates recorded at 60, 72, 84, 96, 108 and 120 h after incubation at 23°C indicates (a) significant difference (P<0.01 by the Student's t-test) between HMGS-OE and the vector (pSa13)-transformed control; (b) indicates significant difference (P<0.01 by the Student's t-test) between OE-wtBjHMGS1 and OE-S359A. Values are mean ±SD (n = 5); bars represent SD. pSa13, vector-transformed control; the two independent lines of OE-wtBjHMGS1 (“401” and “402”) and two independent lines of OE-S359A (“603” and “606”) were tested in seed germination assays. The data represents the average from two transformants. (TIF) [file pone.0098264.s003.tif]

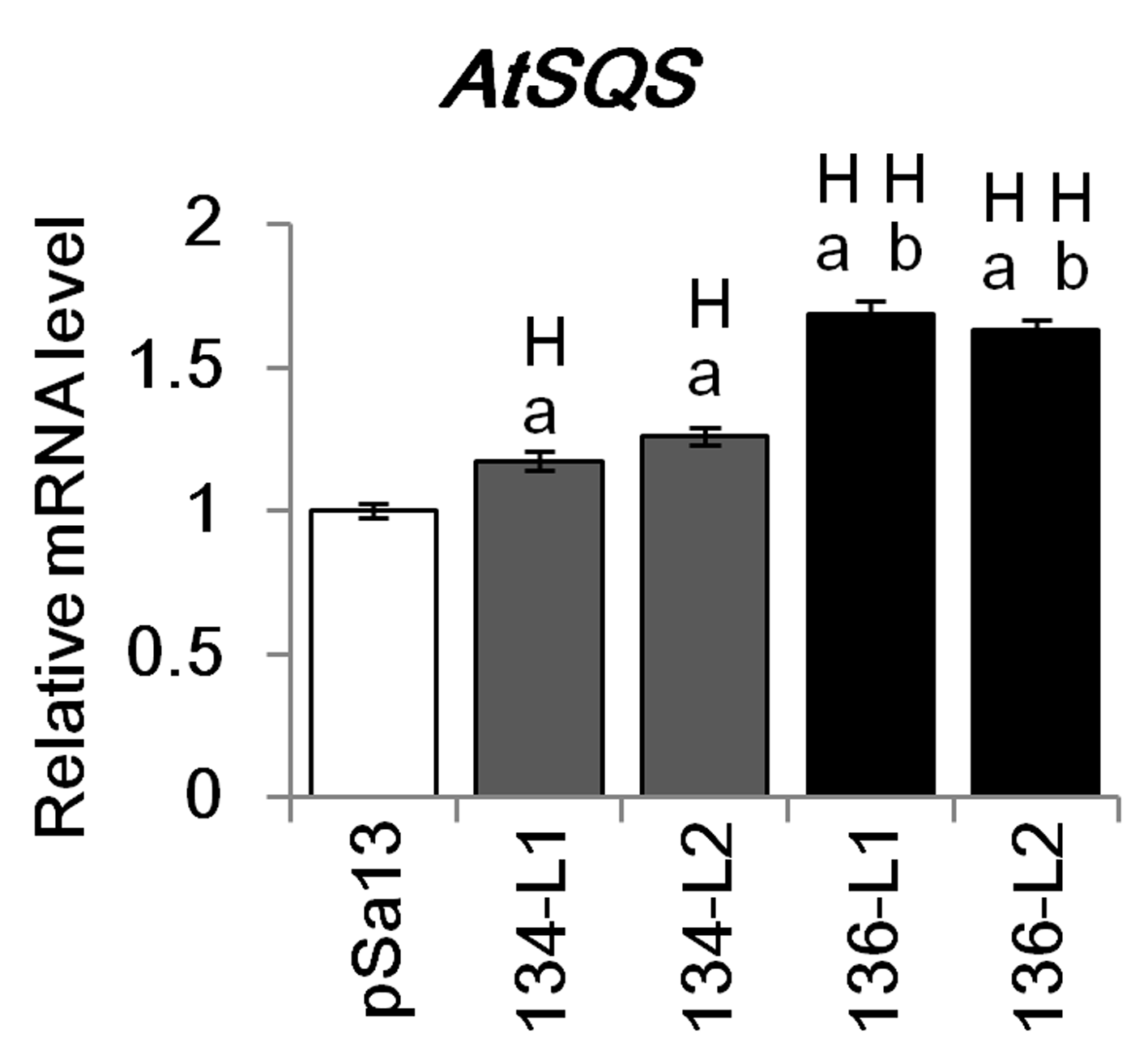

Supplement: Figure S4 — Expression of Arabidopsis SQS by qRT-PCR in 14-d-old HMGS-OE seedlings. Total RNA was extracted from 14-d-old Arabidopsis seedlings of vector (pSa13)-transformed control, two independent lines of OE-wtBjHMGS1 (lines “134-L1” and “134-L2”) and two independent lines of OE-S359A (lines “136-L1” and “136-L2”) previously generated [4]. H, value higher than the control (P<0.01, Student's t-test). Values are means ± SD (n = 3). a indicates significant difference between HMGS-OE and the vector (pSa13)-transformed control; b indicates significant difference between OE-wtBjHMGS1 and OE-S359A. (TIF) [file pone.0098264.s004.tif]
