# Supplementary material for: Transgenic Tobacco Overexpressing Brassica juncea HMG-CoA Synthase 1 Shows Increased Plant Growth, Pod Size and Seed Yield
Source: PLoS One. 2014 May 21;9(5):e98264. doi: 10.1371/journal.pone.0098264 (PMC4029903; doi:10.1371/journal.pone.0098264)
Supplement: Table S1 — Oligonucleotide primers used in this study. Restriction sites are underlined. (DOCX) [file pone.0098264.s005.docx]

**Table S1.** Oligonucleotide primers used in this study

| Gene name | Primer name | Used for | Orientation | Sequence (5’-3’) |
| --- | --- | --- | --- | --- |
| CaMV *35S* promoter | 35S | PCR | Forward | CAATCCCACTATCCTTCGCAAGACC |
| *BjHMGS1* | ML264 | PCR and Southern blot | Reverse | GATCCATAACCAATGGACACTGAGGATCC |
| *BjHMGS1* | ML915 | PCR, sequence | Forward | CATTGCTATGTTGATAGGAC |
| *BjHMGS1* | ML276 | Northern blot | Forward | GGATCCATGGCGAAGAACGTAGGGATATTG |
| *BjHMGS1* | ML860 | Northern and Southern blot | Reverse | GGAGACTGGTTCTCGCAGAGAC |
| *NtHMGR1* | ML1879 | qRT-PCR | Forward | TTGGCATCGGATTTGTTCAG |
| *NtHMGR1* | ML 1880 | qRT-PCR | Reverse | GGCGGCTATCTTCCTCAAT |
| *NtHMGR2* | ML1881 | qRT-PCR | Forward | AGCAGGTGGCGTGAGAAAAT |
| *NtHMGR2* | ML1882 | qRT-PCR | Reverse | CGAACGACTGAACAAATCCG |
| *NtIPI1* | ML1885 | qRT-PCR | Forward | ATCGGGTTTGTTCAGTCGTTC |
| *NtIPI1* | ML1886 | qRT-PCR | Reverse | GCAGGTCCACGACGACTATCT |
| *NtIPI2* | ML1887 | qRT-PCR | Forward | ATTGAGGAGAATGCTCTTGGTG |
| *NtIPI2* | ML1888 | qRT-PCR | Reverse | CTGGTCAACTGGGACATCTTCA |
| *NtFPPS* | ML1889 | qRT-PCR | Forward | CTTCTCCGCAACCACATCAC |
| *NtFPPS* | ML1890 | qRT-PCR | Reverse | GAGGCAGTCTGGAACTCAACC |
| *NtSQS* | ML1678 | qRT-PCR | Forward | AGGAGGTGGAAACAACTGATGA |
| *NtSQS* | ML1679 | qRT-PCR | Reverse | AGAACATACGGCACTTGGGT |
| *NtSMT1-2* | ML1893 | qRT-PCR | Forward | TCCAAATAACGAAGAGCACGAA |
| *NtSMT1-2* | ML1894 | qRT-PCR | Reverse | CAGGCGAATCCTCAGCAAG |
| *NtSMT2-1* | ML1895 | qRT-PCR | Forward | AAACCGCCGATAAAGTCCC |
| *NtSMT2-1* | ML1896 | qRT-PCR | Reverse | TCTACAGCCATTTCTTCGTGAA |
| *NtSMT2-2* | ML1897 | qRT-PCR | Forward | ATCCACGGTATTGAAAGGGG |
| *NtSMT2-2* | ML1898 | qRT-PCR | Reverse | TTTCAGCCTCGTCCACCAC |
| *NtCYP85A1* | ML1899 | qRT-PCR | Forward | AAGAAAAGCCTTCCTCCTGGTA |
| *NtCYP85A1* | ML1900 | qRT-PCR | Reverse | AAAACTCCCAAATCTGGCTCTT |
| *NtACTIN* | ML1951 | qRT-PCR | Forward | TCACAGAAGCTCCTCCTAATCCA |
| *NtACTIN* | ML1952 | qRT-PCR | Reverse | GAGGGAAAGAACAGCCTGAATG |
| *NtGGPPS1* | ML2166 | qRT-PCR | Forward | CTTGTACTGGTAACCCTAATGTTGGA |
| *NtGGPPS1* | ML2167 | qRT-PCR | Reverse | TCCGAGAACTACGGAAGCTTCTA |
| *NtGGPPS2* | ML2168 | qRT-PCR | Forward | TGGAAAACCTCCACAGAGACATT |
| *NtGGPPS2* | ML2169 | qRT-PCR | Reverse | TCAAAGTCAAACTTAGGCAAGATGA |
| *NtGGPPS3* | ML2170 | qRT-PCR | Forward | ATCCGGTGCTCGGCTTCT |
| *NtGGPPS3* | ML2171 | qRT-PCR | Reverse | CTGGTTGATATCACGAATTAGAGTTGT |
| *NtGGPPS4* | ML2172 | qRT-PCR | Forward | GGAGGATTCAATGCTCGGAAA |
| *NtGGPPS4* | ML2173 | qRT-PCR | Reverse | TGGCGCCACCCCACTA |
| *AtSQS* | ML2283 | qRT-PCR | Forward | ATGGCGATTGGAACACTTGC |
| *AtSQS* | ML2284 | qRT-PCR | Reverse | AAGCACCATAGACATCAGCCAT |
| *AtACTIN2* | ML1124 | qRT-PCR | Forward | CCCGCTATGTATGTCGC |
| *AtACTIN2* | ML1125 | qRT-PCR | Reverse | AAGGTCAAGACGGAGGAT |

Restriction sites are underlined
