# Supplementary material for: Transgenic Tobacco Overexpressing Brassica juncea HMG-CoA Synthase 1 Shows Increased Plant Growth, Pod Size and Seed Yield
Source: PLoS One. 2014 May 21;9(5):e98264. doi: 10.1371/journal.pone.0098264 (PMC4029903; doi:10.1371/journal.pone.0098264)
Supplement: Table S2 — Increase (%) of sterol composition in Arabidopsis HMGS-OE seedlings and leaves in comparison to vector (pSa13)-transformed control. (DOCX) [file pone.0098264.s006.docx]

**Table S2. Increase (%) of sterol composition in Arabidopsis HMGS-OE seedlings and leaves in comparison to vector (pSa13)-transformed control**

| **Species** | **Organs** | **Seedlings** | | | | **Leaves** | | | |
| --- | --- | --- | --- | --- | --- | --- | --- | --- | --- |
|  | **Sterols**  **Constructs** | **Campesterol** | **Stigmasterol** | **Sitosterol** | **Total sterol** | **Campesterol** | **Stigmasterol** | **Sitosterol** | **Total sterol** |
| **Arabidopsis** | **134-L1** | 0.9 | 112.5 | 8.7 | 10.7 | 7.0 | -14.3 | 12.7 | 11.3 |
|  | **134-L2** | 1.9 | 125.0 | 8.7 | 11.9 | 9.3 | 2.4 | 19.7 | 15.8 |
|  | **136-L1** | **20.3** | **137.5** | **19.4** | **24.5** | 25.6 | **23.8** | 12.0 | **18.7** |
|  | **136-L2** | **26.2** | **150.0** | **24.0** | **29.0** | 25.6 | **54.8** | 22.7 | **25.9** |
|  | **Elevations of**  **OE-S359A over OE-wtBjHMGS1** | **21.5** | **11.4** | **12.5** | **14.0** | 14.9 | **48.1** | 1.0 | 7.7 |

Two independent lines for each OE genotype were analysed. For Arabidopsis OE-wtBjHMGS1, transformants “134-L1” and “134-L2” were tested. For Arabidopsis OE-S359A, transformants “136-L1” and “136-L2” were tested. Values = [(mean_OEs_ - mean_pSa13_)/mean_pSa13_]*100. The data presented in “Elevations of OE-S359A over OE-wtBjHMGS1” in the table is calculated from an average of two transformants (average of “134-L1” and “134-L2” for OE-wtBjHMGS1 and average of “136-L1” and “136-L2” for OE-S359A). The values of Arabidopsis OE-wtBjHMGS1 and OE-S359A were calculated from our previous published data [4]. Bold font indicates % increase value in OE-S359A which was higher than the corresponding OE-wtBjHMGS1.
